# Gastrointestinal Incomplete Degradation Exacerbates Neurotoxic Effects of PLA Microplastics via Oligomer Nanoplastics Formation

**DOI:** 10.1002/advs.202401009

**Published:** 2024-05-15

**Authors:** Boxuan Liang, Yanhong Deng, Yizhou Zhong, Xiaoqing Chen, Yuji Huang, Zhiming Li, Xiyun Huang, Xiaohong Yang, Jiaxin Du, Rongyi Ye, Hongyi Xian, Yu Feng, Ruobing Bai, Bingchi Fan, Xingfen Yang, Zhenlie Huang

**Affiliations:** ^1^ The Tenth Affiliated Hospital, Southern Medical University (Dongguan People's Hospital) Southern Medical University Dongguan 523059 China; ^2^ NMPA Key Laboratory for Safety Evaluation of Cosmetics Guangdong Provincial Key Laboratory of Tropical Disease Research Department of Toxicology, School of Public Health Southern Medical University Guangzhou 510515 China; ^3^ NMPA Key Laboratory for Safety Evaluation of Cosmetics Guangdong Provincial Key Laboratory of Tropical Disease Research Research Center for Food safety and Health School of Public Health Southern Medical University Guangzhou 510515 China; ^4^ Department of Cardiovascular Surgery Zhujiang Hospital, Southern Medical University Guangzhou 510280 China

**Keywords:** biodistribution, in vivo degradation, mitochondrial calcium uptake family member 3, oligomer nanoplastics, Parkinson's disease

## Abstract

Biodegradable plastics, hailed for their environmental friendliness, may pose unforeseen risks as they undergo gastrointestinal degradation, forming oligomer nanoplastics. Despite this, the influence of gastrointestinal degradation on the potential human toxicity of biodegradable plastics remains poorly understood. To this end, the impact of the murine in vivo digestive system is investigated on the biotransformation, biodistribution, and toxicity of PLA polymer and PLA oligomer MPs. Through a 28‐day repeated oral gavage study in mice, it is revealed that PLA polymer and oligomer microplastics undergo incomplete and complete degradation, respectively, in the gastrointestinal tract. Incompletely degraded PLA polymer microplastics transform into oligomer nanoplastics, heightening bioavailability and toxicity, thereby exacerbating overall toxic effects. Conversely, complete degradation of PLA oligomer microplastics reduces bioavailability and mitigates toxicity, offering a potential avenue for toxicity reduction. Additionally, the study illuminates shared targets and toxicity mechanisms in Parkinson's disease‐like neurotoxicity induced by both PLA polymer and PLA oligomer microplastics. This involves the upregulation of MICU3 in midbrains, leading to neuronal mitochondrial calcium overload. Notably, neurotoxicity is mitigated by inhibiting mitochondrial calcium influx with MCU‐i4 or facilitating mitochondrial calcium efflux with DBcAMP in mice. These findings enhance the understanding of the toxicological implications of biodegradable microplastics on human health.

## Graphical abstract

Polylactic acid (PLA) polymer and oligomer microplastics (MPs) degrade incompletely and completely in mice's gastrointestinal tract, respectively. Incomplete degradation of PLA polymer MPs increases their bioavailability, transforming them into oligomer nanoplastics (NPs). Both types of MPs induce PD‐like neurotoxicity by causing mitochondrial calcium overload in midbrain neurons. Notably, PLA oligomer MPs provoke a more significant neurotoxic response.

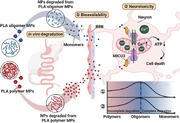

## 1 Introduction

In recent years, a growing imperative to replace widely used fossil‐derived plastics with biodegradable alternatives has emerged.^[^ ^1^ ^]^ However, the assessment of the global shift is predominantly viewed through environmental, ecological, and economic lenses.^[^ ^1^, ^2^, ^3^ ^]^ The impact of this transition on human health remains uncertain. A stark reality is that biodegradable plastics can undergo complete degradation only under specific conditions, including particular temperatures and the requisite presence of microorganisms.^[^ ^4^ ^]^ Indeed, biodegradable plastics often undergo incomplete degradation in the environment, increasing susceptibility to microplastics (MPs) and nanoplastics (NPs) generation compared to traditional fossil‐derived plastics.^[^ ^5^, ^6^ ^]^ The issues of MPs and NPs observed in conventional plastics are similarly pervasive in their biodegradable counterparts.^[^ ^7^ ^]^ Given the widespread biodistribution and organ‐damaging effects observed with MPs and NPs derived from fossil‐derived plastics,^[^ ^8^, ^9^, ^10^, ^11^ ^]^ the potential health hazards posed by those from biodegradable plastics cannot be ignored. Recent revelations highlight toxic effects on invertebrates, zebrafish, and other lower organisms emanating from MPs and NPs in biodegradable plastics.^[^ ^12^, ^13^, ^14^ ^]^ However, a comprehensive exploration of the risks to human health from MPs and NPs derived from biodegradable plastics is imperative.

In contrast to conventional MPs and NPs, the biodegradability inherent in biodegradable MPs and NPs introduces the potential for degradation once inside the human body^[^ ^15^ ^]^ Studies have demonstrated the in vitro digestive degradation of polylactic acid (PLA) MPs, resulting in the formation of oligomer NPs.^[^ ^15^ ^]^ However, whether and how the degradation of biodegradable MPs and NPs within the body may alter their impact on human health remains largely unclear. The degradation products, oligomers, consist of small molecular polymers with 2–40 repeating units, diverging from the larger molecular polymers typically studied in MP and NP research. Their structural diversity suggests potential variations in biological activity compared to their larger counterparts. For instance, PLA oligomers hindered the activation of matrix metallopeptidase 12 in the gut, a phenomenon not observed with PLA polymer MPs and NPs.^[^ ^15^ ^]^ Furthermore, certain polystyrene oligomers have exhibited endocrine‐disrupting properties, in contrast to the inert behavior of polystyrene polymer MPs and NPs.^[^ ^16^ ^]^ Therefore, comprehensive analyses are essential to understand how the digestion and degradation of biodegradable MPs and NPs may influence their biodistribution and biological effects in organisms.

To this end, we investigated the impact of the murine in vivo digestive system on the biotransformation, biodistribution, and toxicity of PLA polymer and PLA oligomer MPs. These models represented biodegradable MPs in their pristine and partially degraded states, mirroring real‐world environmental scenarios that individuals might encounter. Through a 28‐day repeated oral gavage study, we provide a comprehensive panorama of PLA MP biodistribution in mice post‐in vivo digestion, providing foundational data for subsequent toxicity investigations. Acknowledging the brain's pivotal role and susceptibility as a key target organ for MPs and NPs,^[^ ^17^ ^]^ we extensively examined the impact of the in vivo digestive system on PLA MP biotransformation and neurotoxicity. Our findings indicate that incomplete gastrointestinal degradation exacerbates the neurotoxic effects of PLA polymer MPs by releasing oligomer NPs, while complete degradation of PLA oligomer MPs alleviates their neurotoxic effects. Additionally, we identified shared targets and toxicity mechanisms in the neurotoxic effects induced by both PLA polymer and PLA oligomer MPs, contributing to the identification of susceptible populations and the development of practical intervention strategies. This investigation enhances our understanding of the toxicological impacts of biodegradable MPs on human health, laying the groundwork for evaluating the potential benefits or risks associated with the adoption of biodegradable plastics as alternatives to conventional fossil‐derived plastics, particularly concerning human health.

## 2 Results

### 2.1 Degradation of PLA Polymer and Oligomer MPs through In Vivo Digestive System of Mice

**Figure 1:**
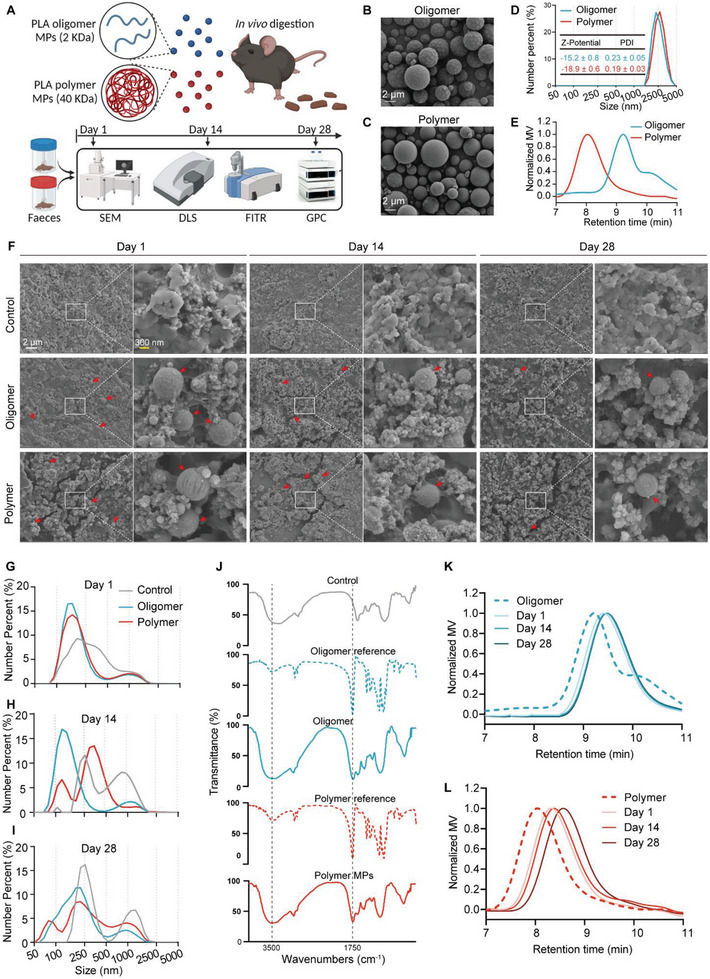
Characterization of PLA particles following in vivo digestion. A) Overview of the experimental design. Representative SEM images depicting B) PLA oligomer MPs and C) PLA polymer MPs before in vivo digestion. D) Z‐potential, PDI, and size distribution of PLA oligomer and polymer MPs prior to in vivo digestion. E) GPC analysis of PLA oligomer and polymer MPs before in vivo digestion. F) Representative SEM images of PLA oligomer and polymer MPs after in vivo digestion for 1, 14, and 28 days. Size distribution of PLA oligomer and polymer MPs after in vivo digestion for G) 1, H) 14, and I) 28 days. J) FTIR spectra of PLA oligomer and polymer MPs after in vivo digestion for 28 days. GPC analysis of K) PLA oligomer and L) polymer MPs after in vivo digestion for 1, 14, and 28 days. FTIR, Fourier‐transform infrared spectroscopy; GPC, gel permeation chromatography; MPs, microplastics; PDI, polydispersity index; PLA, polylactic acid; Z‐potential, zeta potential.

To probe the dynamic degradation profile of PLA polymer and oligomer MPs within the in vivo digestive system, we administered these materials orally to mice for a continuous 28‐day period. To mitigate the influence of particle size on experimental outcomes, we prepared and utilized PLA polymer and oligomer MPs with the same particle size for our experiments. Fecal samples were systematically collected on days 1, 14, and 28 to evaluate the post‐digestion surface morphology, particle size, composition, and molecular weight of the MPs (**Figure** **1**A).

Scanning electron microscopy (SEM) analysis elucidated the spherical morphology of both pristine PLA polymer and oligomer MPs (Figure 1B,C). Dynamic light scattering (DLS) results showcased a consistent size distribution, with both pristine PLA polymer and oligomer MPs measuring ≈2.5 µm in diameter (Figure 1D). The low polymer dispersity index (PDI) and high negative zeta potential reflected the exceptional stability and monodispersity of the polymer and oligomer MPs in double‐distilled water, respectively (Figure 1D). Gel permeation chromatography (GPC) revealed that the molecular weight of pristine PLA polymer MPs was ≈40 kDa, while PLA oligomer MPs exhibited a molecular weight of ≈2 kDa (Figure 1E).

Following one day of in vivo digestion, SEM images of the filtrate from mouse feces treated with either PLA polymer or oligomer MPs revealed spherical particles of varying sizes. The particle size gradually diminished with extended digestion time (Figure 1F). Correspondingly, DLS findings corroborated the SEM results, indicating a substantial presence of nanoparticles with an approximate diameter of 200 nm in the fecal filtrate after one day of in vivo digestion for both PLA polymer and oligomer MPs (Figure 1G). By day 14 of digestion, smaller nanoparticles, ≈100 nm in diameter, were evident in the fecal filtrate (Figure 1H). By day 28 of digestion, even smaller nanoparticles with diameters below 100 nm were detected (Figure 1I). Notably, the digestion rate of PLA oligomer MPs outpaced that of PLA polymer MPs, as evidenced by the higher proportion of nanoparticles digested from PLA oligomer MPs compared to PLA polymer MPs (Figure 1G–I). Crucially, we observed that the number of nanoparticles with diameters below 100 nm, resulting from the digestion of PLA oligomer MPs, was lower than those derived from PLA polymer MPs at day 28 (Figure 1I). This phenomenon may be attributed to the complete degradation of a portion of nanoparticles originating from PLA oligomer MPs. The Fourier‐transform infrared (FTIR) spectrum of the fecal filtrate exhibited distinct peaks at 3500 cm^−1^ (infrared absorption peak of ─OH) and 1750 cm^−1^ (infrared absorption peak of ─COOH), confirming the presence of PLA in the fecal filtrate (Figure 1J; Figure S1A,B, Supporting Information). Furthermore, GPC analysis revealed a decrease in the molecular weights of both PLA polymer and oligomer MPs following one day of in vivo digestion (Figure 1K,L). The degradation of PLA oligomer MPs reached a plateau more rapidly, approaching approximately 900 Da after just one day of in vivo digestion (Figure 1K). In contrast, the degradation of PLA polymer MPs exhibited an initially slow rate during the initial 14 days of digestion, with an acceleration becoming evident as digestion time progressed (Figure 1L). Collectively, our findings underscore that PLA polymer and oligomer MPs undergo degradation into nanoparticles within an in vivo digestive system, with the degradation rate of PLA oligomer MPs surpassing that of PLA polymer MPs.

### 2.2 Biodistribution of Particles Digested from PLA Polymer and Oligomer MPs in Mice

**Figure 2:**
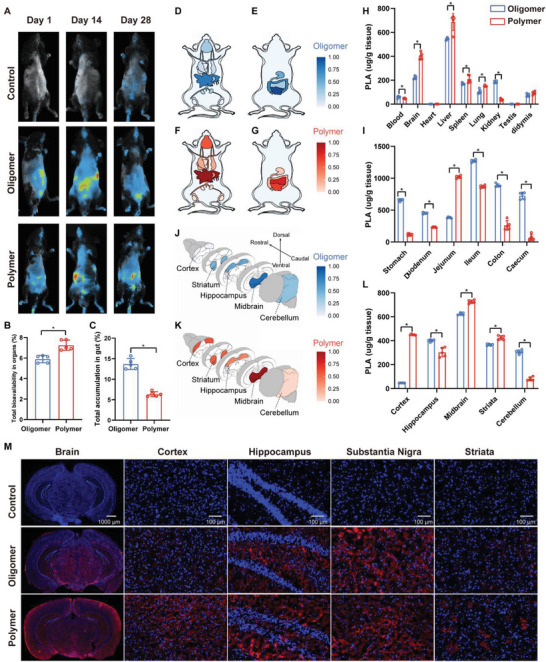
Biodistribution of PLA particles following in vivo digestion. A) Dynamic biodistribution following expo sure to PLA oligomer and polymer MPs. B) Total bioavailability of PLA particles in organs and C) total accumulation of PLA particles in the gastrointestinal tract after 28 consecutive days of exposure to PLA oligomer and polymer MPs. Summarized diagrams illustrating D) organ biodistribution and E) gastrointestinal accumulation of PLA particles after 28 consecutive days of exposure to PLA oligomer MPs. Summarized diagrams illustrating F) organ biodistribution and G) gastrointestinal accumulation of PLA particles after 28 consecutive days of exposure to PLA polymer MPs. Quantitative results of H) organ biodistribution and I) gastrointestinal accumulation. Summarized diagrams illustrating the biodistribution of PLA particles in brain regions after 28 consecutive days of exposure to J) PLA oligomer and K) polymer MPs for 28 consecutive days. L) Quantitative results and M) representative images depicting biodistribution in individual brain regions. Statistical analyses are determined by Student's *t*‐test; ^*^ *p* < 0.05. MPs, microplastics; PLA, polylactic acid.

To dynamically monitor the biodistribution of particles resulting from the in vivo digestion of PLA polymer and oligomer MPs in mice over a 28‐day exposure period, we utilized an in vivo fluorescence imaging system to track fluorescence particles on days 1, 14, and 28. Preliminary assessments ensured negligible fluorescence leakage from each type of PLA MPs using an in vitro‐digested system (Figure S2, Supporting Information). Our observations revealed that particles derived from both PLA polymer and oligomer MPs underwent temporal movement and accumulation in the digestive system of mice during the 28‐day exposure. However, the in vivo fluorescence imaging system detected no other fluorescent signals in any other organs (**Figure** **2**A). Subsequent examination of the biodistribution of particles derived from PLA polymer and oligomer MPs in organs and their accumulation in intestine segments was conducted following homogenization. The established standard curves for each tissue and blood demonstrated a linear correlation between fluorescence intensities and concentrations of PLA polymer and oligomer MPs (Figures S3 and S4, Supporting Information). Notably, particles resulting from PLA polymer MPs exhibited higher total bioavailability compared to those from PLA oligomer MPs (7.3% vs 5.9%) (Figure 2B), while particles derived from PLA polymer MPs showed lower total accumulation compared to those from PLA oligomer MPs (6.3% vs 13.7%) (Figure 2C). Detailed biodistribution of particles resulting from PLA polymer and oligomer MPs in each organ and their accumulation in each intestine segment in mouse models is summarized in diagrams (Figure 2D–G). Specifically, particles resulting from both PLA polymer and oligomer MPs were detected in the blood, brain, liver, spleen, lungs, kidneys, and epididymis, while being absent in the heart and testis (Figure 2H). The biodistribution of particles derived from PLA polymer MPs in the brain, liver, spleen, and lungs exceeded that from PLA oligomer MPs, while the biodistribution of particles derived from PLA polymer MPs in the blood and kidneys was lower than that from PLA oligomer MPs (Figure 2H). Furthermore, the accumulation of particles derived from PLA polymer MPs in all intestine segments was lower than that from PLA oligomer MPs, except for the jejunum (Figure 2I).

Significantly, upon homogenizing each brain partition, we observed distinct variations in the biodistribution characteristics of particles degraded from PLA polymer and oligomer microparticles (MPs) across key brain regions, encompassing the cortex, hippocampus, striatum, midbrain, and cerebellum. Among these, the distribution of particles derived from both PLA polymer and oligomer MPs was most prominent in the midbrain (Figure 2J–L). Specifically, particles from PLA polymer MPs exhibited a higher distribution in the cortex, striatum, and midbrain, whereas those from PLA oligomer MPs displayed a preference for the hippocampus and cerebellum (Figure 2J–L). This biodistribution pattern in each brain region was further validated through fluorescence microscopy (Figure 2M). Evans blue staining demonstrated that, following 28 days of exposure to PLA polymer or oligomer MPs, the integrity of the blood‐brain barrier (BBB) remained uncompromised (Figure S5A,B, Supporting Information). Expression analysis of tight junction protein‐related genes revealed only slight decreases in the hippocampus, no significant changes in the striatum, and even an increase in the cortex and midbrain (Figure S5C–N, Supporting Information). These results indicated that the particles distributed in the brain due to PLA digestion are nanoparticles, as they exhibit the capability to freely penetrate the BBB. Our findings once again emphasize that PLA polymer and oligomer MPs undergo degradation into nanoparticles within the in vivo digestive system of mice. Furthermore, the biodistribution of nanoparticles released from PLA polymer MPs after in vivo digestion is higher than that from PLA oligomer MPs in the brains.

### 2.3 Nanoplastics Released from Digested PLA Polymer and Oligomer MPs Induce Parkinson's Disease (PD)‐like Neurodegeneration in Mice

**Figure 3:**
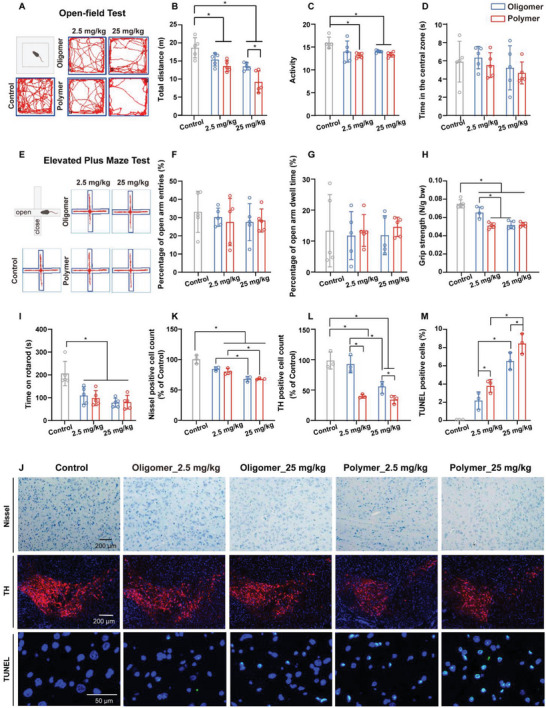
Neurotoxicity induced by PLA oligomer and polymer MP exposure. A) Representative track images in the open field test. Quantitative analysis of B) total distance, C) activity, and D) time spent in the central zone during the open field test. E) Representative track images in the elevated plus maze test. Quantification of percentage F) open arm entries and G) open arm dwell time in the elevated plus maze test. H) Grip strength in the grip strength test. I) Latency time in the rotarod test. J) Representative images of Nissl staining, TH staining, and TUNEL staining in mouse midbrains. Relative percentages of positively stained cells in the midbrain: K) Nissl staining, L) TH staining, and M) TUNEL staining. Statistical analyses are determined by ANOVA, followed by Tukey's multiple comparison tests; ^*^ *p* < 0.05. ANOVA, analysis of variance; MPs, microplastics; PLA, polylactic acid. TH, tyrosine hydroxylase; TUNEL, TdT‐mediated dUTP nick‐end labeling.

We monitored the body weight and organ coefficients of each animal as indicators of general toxicity. However, we observed that neither PLA polymer nor oligomer MPs resulted in significant changes in animal body weight or organ coefficients (Table S1, Supporting Information). To further investigate the potentially detrimental effects of brain‐distributed NPs degraded from PLA polymer and oligomer MPs, we evaluated the behavioral abilities and depression levels of mice following a 28‐day exposure to PLA polymer or oligomer MPs. The open‐field test revealed a dose‐dependent decrease in movement distances for mice exposed to PLA polymer or oligomer MPs. Notably, mice exposed to PLA polymer MPs exhibited a more pronounced reduction in movement distance compared to those exposed to oligomer MPs (**Figure** **3**A,B). Furthermore, the activity of the mice diminished upon exposure to PLA polymer or oligomer MPs (Figure 3C). However, no changes were observed in the mice's resident time in the central zone during exposure to PLA polymer or oligomer MPs (Figure 3D). Similarly, no alterations were noted in the number and time of entry into the open arm during the elevated plus maze test for mice exposed to PLA polymer or oligomer MPs (Figure 3E–G). These findings suggest that NPs degraded from PLA polymer and oligomer MPs impacted the behavioral abilities of the mice without affecting their emotional states.

To further substantiate the neurobehavioral deficits induced by NPs degraded from PLA polymer and oligomer MPs, we conducted grip strength and rotarod tests to evaluate myodynamia and coordination, respectively. The mice displayed diminished grip strength and a reduced latency for falling off the rod following exposure to PLA polymer or oligomer MPs (Figure 3H,I). In light of these distinct functional impairments, we proceeded to investigate the impact of NPs degraded from PLA polymer and oligomer MPs on various brain regions. Nissl staining on nerve cells unveiled a dose‐dependent decrease in Nissl bodies in the substantia nigra pars compacta (SNc) for mice exposed to PLA polymer or oligomer MPs, with no significant changes observed in the cortex, striatum, and hippocampus (Figure 3J,K; Figure S6, Supporting Information). TH staining on dopaminergic neurons revealed a dose‐dependent reduction in TH‐positive cells in the SNc for mice exposed to PLA polymer or oligomer MPs, while no significant change was observed in the striatum (Figure 3J,L; Figure S7A,C, Supporting Information). Notably, mice exposed to PLA polymer MPs exhibited a more pronounced reduction in TH‐positive cells compared to those exposed to oligomer MPs (Figure 3J,L). Further examination using TUNEL staining on apoptotic cells demonstrated a dose‐dependent increase in TUNEL‐positive cells in the SNc for mice exposed to PLA polymer or oligomer MPs, without significant changes in the cortex, striatum, and hippocampus (Figure 3J,M; Figure S7B,D–F, Supporting Information). Significantly, mice exposed to PLA polymer MPs exhibited a more pronounced increase in TUNEL‐positive cells compared to those exposed to oligomer MPs (Figure 3J,M). In summary, our findings indicate that post in vivo digestion, PLA polymer, and oligomer MPs induce specific neuronal death in the SNc of mice, compromising motor neural function and leading to PD‐like neurodegeneration. Notably, PLA polymer MPs exert a more pronounced neurotoxic effect compared to oligomer MPs.

### 2.4 PLA Oligomer and Polymer MPs Induce Mitochondrial Calcium Overload by Upregulating mitochondrial calcium uptake family member 3 (MICU3) in the Midbrain of Mice

**Figure 4:**
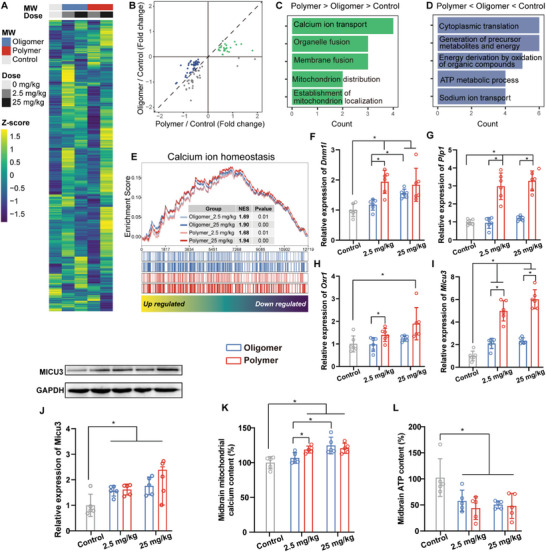
MICU3‐mediated mitochondrial calcium overload in the midbrain of mice is induced by PLA oligomer and polymer MP exposure. A) Transcriptome profiles of midbrains after 28 consecutive days of exposure to PLA oligomer and polymer MPs. B) Scatter diagram depicting DEGs in midbrains after 28 consecutive days of exposure to PLA oligomer and polymer MPs. GO analysis of the DEGs in the order of C) PLA polymer MPs > PLA oligomer MPs > control, and D) PLA polymer MPs < PLA oligomer MPs < control. E) GSEA of transcriptome data. Validation of mRNA expression through qPCR for F) *Dnm1l*, G) *Plp1*, H) *Oxr1*, and I) *Micu3* in midbrains. J) Protein expression of MICU3 in midbrains. K) Relative mitochondrial calcium concentration and L) relative ATP content in midbrains after 28 consecutive days of exposure to PLA oligomer and polymer MPs. Statistical analyses are determined by ANOVA, followed by Tukey's multiple comparison tests; ^*^ *p* < 0.05. ANOVA, analysis of variance; ATP, Adenosine triphosphate; DEGs: differentially expressed genes; Dnm1l, dynamin 1 like; GSEA: gene set enrichment analysis; GO: gene ontology; Micu3, mitochondrial calcium uptake family member 3; MPs, microplastics; Oxr1, oxidation resistance 1; NES, normalized enrichment score; PLA, polylactic acid; Plp1, proteolipid protein 1; qPCR, quantitative PCR.

We subsequently performed a transcriptomic analysis to unravel the mechanisms underlying the PD‐like neurodegeneration induced by the NPs degraded from PLA polymer and oligomer MPs in mice (**Figure** **4**A). Analysis of differentially expressed genes (DEGs) in the high dose groups revealed a consistent trend, where almost all DEGs induced by the NPs degraded from PLA polymer or oligomer MP exhibited uniform changes, either increased or decreased (Figure 4B). This indicates that the toxic mechanisms triggered by the NPs degraded from PLA polymer or oligomer MPs in the mouse midbrain are uniform, with variations observed only in the magnitude of the alterations.

Considering the stronger neurotoxic effect exhibited by the NPs degraded from PLA polymer MPs compared to those from oligomer MPs, we prioritized the selection of DEGs in the order of PLA polymer MPs > PLA oligomer MPs > control, or PLA polymer MPs < PLA oligomer MPs < control. Subsequently, we conducted a Gene Ontology (GO) analysis. The results revealed that progressively upregulated DEGs were primarily associated with calcium ion transport (Figure 4C), while the downregulated DEGs were mainly involved in adenosine triphosphate (ATP) metabolic processes (Figure 4D). Consistent findings across all genes and groups were confirmed through Gene Set Enrichment Analysis (GSEA), showing a dose‐response elevation of normalized enrichment score (NES) in the calcium ion homeostasis pathway (Figure 4E).

To validate these results, we examined the mRNA expression of four DEGs associated with calcium ion transport—namely *Dnm1l*, *Plp1*, *Oxr1*, and *Micu3*—in various brain regions using quantitative polymerase chain reaction (qPCR). No significant increase in the expression of these four genes was observed in the cortex (Figure S8A–D, Supporting Information), hippocampus (Figure S8E–H, Supporting Information), and striatum (Figure S8I–L, Supporting Information). Significantly, we observed a substantial upregulation of the four DEGs in the midbrain of mice following exposure to both PLA polymer and PLA oligomer MPs (Figure 4F–I). Notably, *Micu3* demonstrated the highest fold change among them (Figure 4F–I). The protein expression of MICU3 was also upregulated (Figure 4J).

Considering the aforementioned observations and acknowledging the pivotal role of MICU3 in mitochondrial calcium transport, we propose that the NPs degraded from PLA polymer and oligomer MPs induce mitochondrial calcium overload by upregulating MICU3 in the midbrain of mice. This, in turn, disrupts and diminishes ATP metabolism, ultimately culminating in PD‐like neurodegeneration. Our results substantiate this hypothesis, revealing that following 28 days of PLA polymer or oligomer MP exposure, although there were no observable changes in the morphology of mitochondria in the midbrain of mice (Figure S9, Supporting Information), there was an elevation in mitochondrial calcium concentration (Figure 4K) and a reduction in tissue ATP content (Figure 4L) in the midbrain of mice. Moreover, we did not observe any alterations in mitochondrial calcium concentration (Figure S8M–O, Supporting Information) and tissue ATP content (Figure S8P–R, Supporting Information) in other brain regions of mice after 28 days of PLA polymer or oligomer MP exposure.

### 2.5 Inhibiting Micu3 Expression Mitigated PLA MP‐Induced Neurotoxicity in Neural Cells

**Figure 5:**
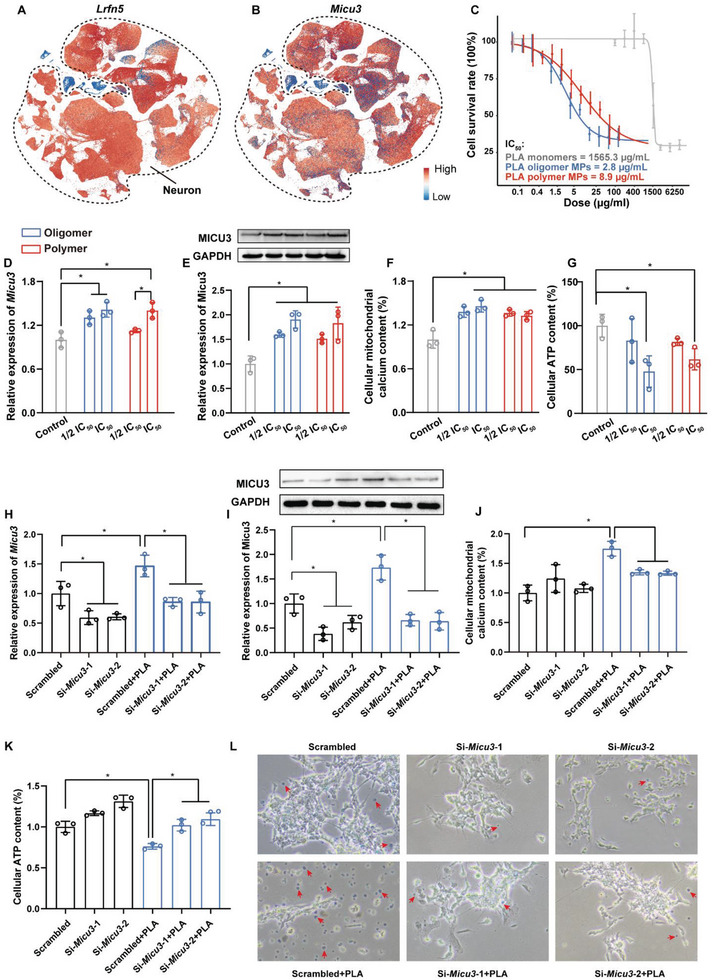
Inhibiting *Micu3* expression mitigated PLA MP‐induced neurotoxicity in differentiated SH‐SY5Y cells. *t*‐SNE plot showing A) the canonical neuron marker gene *Lrfn5* and B) the expression of our target gene *Micu3* in a single‐cell RNA atlas of the mouse brain from the ALLEN BRAIN MAP.^[^ ^18^ ^]^ C) IC_50_ values for PLA oligomer and polymer MPs in differentiated SH‐SY5Y cells after 24‐hour exposure, were determined using a cell viability assay. D) mRNA and E) protein expression of MICU3 in differentiated SH‐SY5Y cells after 24‐h exposure to PLA oligomer and polymer MPs. F) Relative mitochondrial calcium concentration and G) relative ATP content in differentiated SH‐SY5Y cells after 24‐h exposure to PLA oligomer and polymer MPs. H) mRNA and I) protein expression of MICU3 in differentiated SH‐SY5Y cells after 24‐h exposure to PLA oligomer MPs while inhibiting *Micu3* expression. J) Relative mitochondrial calcium concentration, K) relative ATP content, and L) cell viability in differentiated SH‐SY5Y cells after 24‐h exposure to PLA oligomer MPs while inhibiting *Micu3* expression. Statistical analyses are determined by ANOVA, followed by Tukey's multiple comparison tests; ^*^ *p* < 0.05. ANOVA, analysis of variance; ATP, adenosine triphosphate; IC_50_, half maximal inhibitory concentration; *Lrfn5*, leucine‐rich repeat and fibronectin type III domain containing 5; *Micu3*, mitochondrial calcium uptake family member 3; MPs, microplastics; PLA, polylactic acid.

By employing Lrfn5 as the neuronal marker, we identified neural cells in a single‐cell RNA atlas of the mouse brain from the ALLEN BRAIN MAP (**Figure** **5**A).^[^ ^18^ ^]^ Our analysis revealed specific expression of *Micu3* in neural cells (Figure 5B), implying that the PLA NP‐induced upregulation of *Micu3* in the midbrain of mice primarily occurs in neural cells. Consequently, we utilized a dopamine‐neuron‐like cell line, differentiated SH‐SY5Y neural cells, in vitro to elucidate the underlying mechanism of *Micu3* in PLA NP‐induced neurotoxicity. The CCK‐8 assay demonstrated that the cellular toxicity of PLA monomers was significantly lower than both types of MPs. Notably, PLA oligomer MPs exhibited higher cellular toxicity than PLA polymer MPs, with LD_50_ values of 2.8 and 8.9 µg mL^−1^, respectively (Figure 5C). For subsequent in vitro experiments, we employed half of the LD_50_ values for both PLA oligomer and polymer MPs as low‐dose, and the LD_50_ as high‐dose exposure concentrations. Both PLA oligomer and polymer MPs induced upregulation of Micu3 mRNA and protein expression in differentiated SH‐SY5Y cells (Figure 5D,E). Meanwhile, PLA oligomer and polymer MP exposure led to an increase in mitochondrial calcium concentration and a decrease in cellular ATP concentration (Figure 5F,G). Our results indicate that both PLA oligomer and polymer MPs induce neurotoxicity in differentiated SH‐SY5Y neural cells, with PLA oligomer MPs exhibiting a stronger toxic effect.

Our previous transcriptomic findings had already indicated a consistent mechanism of neurotoxicity induced by PLA oligomer and polymer MPs. Subsequently, we chose the more pronouncedly in vitro toxic PLA oligomer MPs for further experiments. Differentiated SH‐SY5Y neural cells were separately transfected with scrambled siRNA and two different *Micu3* siRNAs. Upon successfully inhibiting the upregulation of *Micu3* expression induced by PLA MPs (Figure 5H,I), we observed an attenuation of the increase in mitochondrial calcium concentration and the decrease in cellular ATP concentration caused by PLA MPs (Figure 5J,K). Moreover, inhibiting *Micu3* expression mitigated the cell death induced by PLA MPs (Figure 5L). Our findings suggest that PLA MPs induce neurotoxicity in neural cells by upregulating *Micu3* expression, leading to an increase in mitochondrial calcium concentration, a subsequent decrease in cellular ATP concentration, and ultimately neural cell death.

### 2.6 Inhibiting Mitochondrial Calcium Influx or Facilitating Mitochondrial Calcium Efflux Mitigated PLA MP‐induced PD‐like Neurodegeneration in Mice

**Figure 6:**
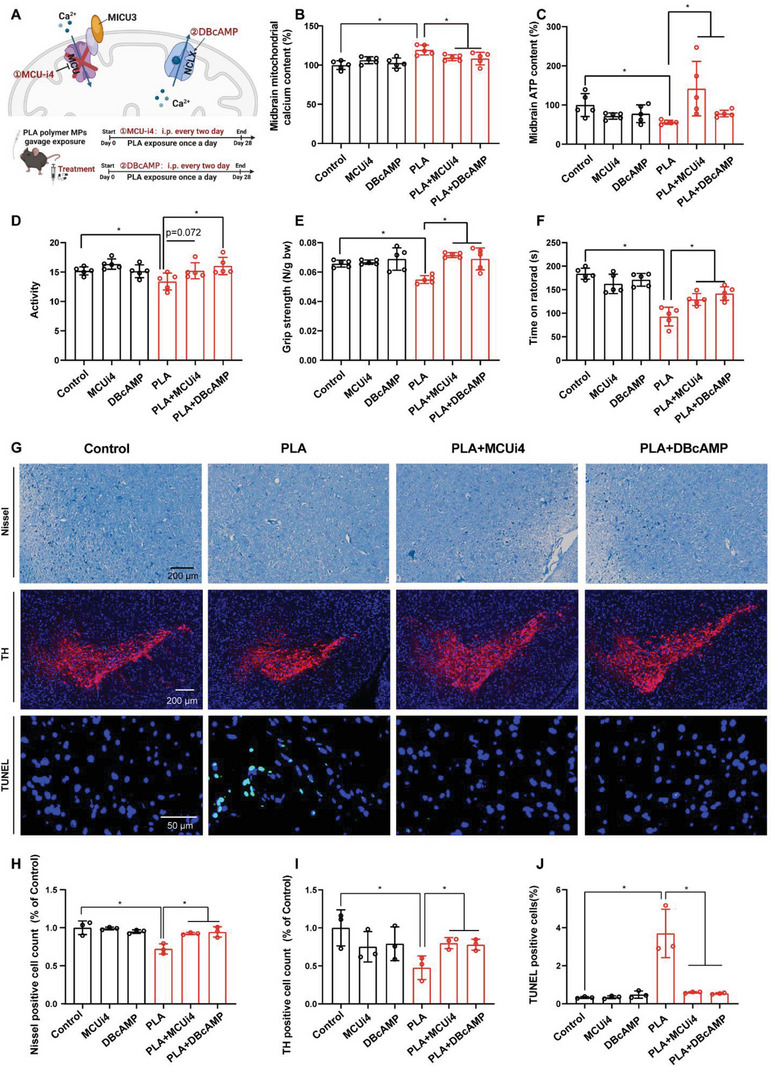
Stabilizing mitochondrial calcium levels ameliorates PLA MP‐induced PD‐like neurodegeneration in mice. A) Treatment strategy and experimental design overview. B) Relative mitochondrial calcium concentration and C) relative ATP content in midbrains after consecutive 28 days of exposure to PLA polymer MPs while treated with MCU‐i4 or DBcAMP. D) Activity levels in the open field test. E) Grip strength assessed in the grip strength test. F) Latency time measured in the rotarod test. G) Representative images of Nissl staining, TH staining, and TUNEL staining in mouse midbrains. Relative percentages of positive staining in midbrain cells for H) Nissl staining, I) TH staining, and J) TUNEL staining. Statistical analyses are determined by ANOVA, followed by Tukey's multiple comparison tests; ^*^ *p* < 0.05. ANOVA, analysis of variance; *Micu3*, mitochondrial calcium uptake family member 3; MPs, microplastics; PLA, polylactic acid. TH, tyrosine hydroxylase; TUNEL, TdT‐mediated dUTP nick‐end labeling.

Subsequently, we exposed mice to 25 mg kg^−1^ PLA polymer MPs for consecutive 28 days while concurrently administering MCU‐i4 (mitochondrial calcium uniporter inhibitor; MCU inhibitor) or DBcAMP (Na^+^/Ca^2+^ exchanger agonist; NCLX agonist) through intraperitoneal injection for treatments (**Figure** **6**A). The objective was to inhibit mitochondrial calcium influx or promote mitochondrial calcium efflux, aiming to further confirm that the PD‐like neurodegeneration induced by PLA polymer MPs in mice is attributed to mitochondrial calcium overload in midbrain neurons. As a result, both MCU‐i4 and DBcAMP treatments successfully restored mitochondrial calcium overload and the decrease in cellular ATP induced by PLA polymer MPs to normal levels in the midbrain of mice (Figure 6B,C). Furthermore, both treatments significantly alleviated PLA MP‐induced PD‐like neurodegeneration, manifested by the amelioration of motor and coordination disorders, as evidenced by restored locomotor activity levels (Figure 6D), improved grip strength (Figure 6E), and increased latency time for falling off the rod (Figure 6F). They also mitigated the loss of neurons, especially dopaminergic neurons, in the SNc caused by PLA MPs, as seen in Nissl, TH, and TUNEL staining (Figure 6G–J; Figure S10, Supporting Information).

## 3 Discussion

The adoption of biodegradable plastics as a substitute for conventional ones is increasingly regarded as a solution to the pervasive “plastic problem”.^[^ ^1^ ^]^ However, the decision to embrace this substitution is primarily motivated by environmental and economic factors. Whether the transition to biodegradable plastics can benefit human health in the current plastic‐polluted environment requires further exploration. Previous studies have demonstrated that PLA MPs undergo digestion by gastric and intestinal lipases, releasing degraded oligomer NPs that are bioavailable and capable of inducing acute inflammation in the gut.^[^ ^15^ ^]^ However, the impact of PLA MP digestion and degradation on its biodistribution and biological effects in various organs of humans remains unclear. Our present study provides insights into the fact that in vivo digestion can completely degrade specific PLA oligomer MPs, consequently reducing their biodistribution in organs. However, it is noteworthy that PLA oligomer particles resulting from the incomplete degradation of PLA polymer MPs may exhibit heightened neurotoxicity. Consequently, the influence of biodegradable MPs on human health is contingent on the extent of their degradation through in vivo digestion. Complete degradation can mitigate their impact on human health, while incomplete degradation may exacerbate the harmful biological effects of biodegradable MPs.

The toxicological properties of NPs and MPs primarily stem from either their adsorbed chemical additives or the particles themselves.^[^ ^19^, ^20^ ^]^ In the case of biodegradable NPs and MPs, due consideration must also be given to their chemical monomers, considering their degradability.^[^ ^21^ ^]^ In our study, we posit that the primary source of toxicity from both PLA polymer and oligomer MPs is predominantly associated with the intrinsic toxicity of the particles themselves. This assertion is based on our findings, which demonstrate that the PLA monomer, lactic acid, while serving as an endogenous compound influencing energy metabolism,^[^ ^22^ ^]^ exhibits limited toxicity to neural cells at equivalent concentrations of PLA polymer and oligomer MPs. Furthermore, the use of virgin PLA MPs in our experiments eliminates the possibility of toxicity arising from chemical additives. This observation aligns with similar findings in Daphnia magna.^[^ ^23^ ^]^ Therefore, the crucial aspect in assessing the impact of in vivo digestion on the toxicity effects of PLA polymer and oligomer MP exposure lies in understanding how in vivo digestion influences the inherent nature of these particles themselves.

The pivotal findings of our study underscore the vulnerability of PLA polymer MPs to incomplete degradation in the gastrointestinal tract, resulting in the formation of oligomers and nanoparticles. Notably, particles >200 nm in size or with molecular weights exceeding 10 kDa are impeded from freely entering the brain by the BBB.^[^ ^24^, ^25^ ^]^ Given that our results demonstrate the integrity of this barrier under the current exposure conditions in mice, the penetration of PLA polymer MPs into the brain and their subsequent toxicity can solely be attributed to their digestion and degradation into smaller‐sized PLA oligomer NPs. Furthermore, our observations reveal that, even when controlling for size factors, the neurotoxicity of PLA oligomer MPs surpasses that of PLA polymer MPs, possibly due to the facilitated penetration of oligomers through the cell membrane.^[^ ^26^ ^]^ A similar phenomenon has been previously observed in intestinal cells in a different study.^[^ ^15^ ^]^ Hence, the incomplete degradation of PLA polymer MPs in the gastrointestinal tract not only increases their bioavailability but also heightens their toxicity, amplifying their overall toxic effects. This is consistent with smaller‐sized MPs and NPs typically exhibiting broader biodistribution in organs and more severe toxicity.^[^ ^27^, ^28^, ^29^, ^30^ ^]^

Conversely, PLA oligomer MPs and NPs can undergo further complete degradation in the gastrointestinal tract, as evidenced by both our in vivo and previous in vitro digestion results.^[^ ^15^ ^]^ This thorough degradation serves to limit the biodistribution of PLA particles in organs. Furthermore, our in vitro study indicates that the toxicity of PLA oligomer MPs on neural cells diminishes as they continue to degrade into monomers. This observation aligns with the established understanding that toxic effects become apparent only when pathological proteins aggregate from monomers to oligomers.^[^ ^31^, ^32^ ^]^ Thus, considering both biodistribution and toxicity, the susceptibility of PLA MPs to degradation in the gastrointestinal tract presents a double‐edged sword for human health. Incomplete degradation of PLA polymer MPs heightens the bioavailability and toxicity of PLA particles, thereby amplifying the overall toxic effects associated with PLA MPs. Conversely, the complete degradation of PLA oligomer MPs reduces the bioavailability of PLA particles and weakens their toxicity, ultimately mitigating the overall toxic effects induced by PLA MPs. Therefore, ensuring the thorough degradation of PLA MPs into small molecules within the in vivo digestive system, rather than incomplete degradation into oligomers, is crucial for mitigating their impact on human health.

Another intriguing observation from our study is that, despite alterations in the toxicity intensity of PLA MPs caused by in vivo degradation, the neurotoxicity mechanisms of PLA MPs remain unchanged during the degradation process. Our findings reveal that both PLA polymer and oligomer MPs induce PD‐like neurotoxicity in mice by upregulating the expression of MICU3 in the midbrains, subsequently leading to mitochondrial calcium overload in neurons. This may partially explain why in vivo degradation only modifies the toxicity intensity of PLA MPs. Although neuron mitochondria are typically targeted by MPs, the specific molecular targets vary depending on their polymer types. For instance, PS NPs induce PD‐like neurodegeneration by promoting excessive autophagy of neuronal mitochondria through the AMPK/ULK1 pathway.^[^ ^33^, ^34^ ^]^ In contrast, weathered MPs composed of propylene and ethylene induce anxiety‐like behaviors, accompanied by a significant decrease in mitochondrial respiratory chain complex II and IV.^[^ ^35^ ^]^ Changes in physical properties, such as particle size and molecular weight, due to degradation may only modify the intensity of particle toxicity. In comparison, the polymer type is the key factor determining the toxicity endpoint and mechanism, emphasizing the importance of polymer type over physical properties like size and molecular weight in determining the toxicity of MPs. This finding simplifies the exploration of the toxicity and mechanisms of the same type of biodegradable MPs with different sizes and molecular weights.

According to our results, the key to reducing the toxic effects of PLA MPs lies in ensuring their complete degradation in the gastrointestinal tract before absorption rather than incomplete degradation. We found that the lower the molecular weight of PLA MPs, the more easily they can be degraded by the in vivo digestive system. This characteristic mirrors the findings in another structurally similar material, PLGA.^[^ ^36^ ^]^ Mechanistically, this phenomenon may be attributed to the reduced steric hindrance of lower molecular weight particles, facilitating easier degradation.^[^ ^37^ ^]^ The complete degradation of PLA MPs under normal digestive conditions depends largely on their initial molecular weight, with our observations suggesting that only when the initial exposed molecular weight is below 2 kDa can some PLA MPs be fully degraded by the mouse digestive system. Given that the current molecular weight of PLA MPs in the environment exceeds this threshold, we conclude that the digestive system only partially degrades the PLA MPs ingested from the environment under prevailing conditions, consequently exacerbating their biodistribution and toxicity effects on humans. Our findings underscore the importance of degrading discarded PLA in the environment not only from an environmental perspective but also from a human health standpoint. Furthermore, for optimizing the design of biodegradable polymers used in direct contact with humans, it is advisable to prioritize the use of lower molecular weight materials while ensuring their functionality.

The levels of PLA MPs in the environment have not been adequately characterized, and the realistic human exposure levels remain unclear.^[^ ^38^ ^]^ However, given the escalating severity of global plastic pollution, it is imperative not only to investigate the potential hazards of PLA MPs at current realistic levels, but also to consider higher concentrations that may emerge in the future.^[^ ^2^, ^39^ ^]^ Based on current data indicating human ingestion of approximately 0.1–5.0 grams of MPs weekly,^[^ ^40^ ^]^ equivalent to roughly 0.2–10 mg kg^−1^ body weight/day (assuming an adult body weight of 70 kg), our study involved doses of 2.5 mg kg^−1^ PLA MPs to simulate current human exposure levels and 25 mg kg^−1^ PLA MPs to identify potential hazards.

We have exclusively examined PLA as a representative of biodegradable plastics. Caution is warranted when extrapolating our findings to other biodegradable plastics. Furthermore, our current study utilized pristine PLA microparticles. However, plastic products often contain chemical additives during manufacturing, and whether this affects the in vivo digestion rate of biodegradable plastics remains to be elucidated. Additionally, the impact of in vivo digestion on the toxicity of chemical additives adsorbed to biodegradable plastics is also unclear. Nevertheless, we posit that by comparing other biodegradable plastics with PLA concerning the sources of MPs' toxicity, the rate of in vivo degradation, and the toxicity of their degradation products, including oligomers and monomers, some initial insights into the toxicity of other biodegradable plastics can be gained. Given the substantial impact of gastrointestinal digestion on the toxicity of biodegradable plastics, further exploration should delve into the factors influencing the degradation of biodegradable plastics in the gastrointestinal tract. This exploration would help propose methods to promote the complete degradation of biodegradable plastics in the gastrointestinal tract, thereby reducing their potential harm to human health. Furthermore, although we have initially elucidated the neurotoxic mechanisms of PLA polymer and oligomer MPs, their toxicity to other organs and tissues remains to be clarified. Moreover, the mechanisms underlying the distinct biodistribution and toxicity intensity of PLA polymer and oligomer microplastics also require further elucidation.

**Scheme 1:**
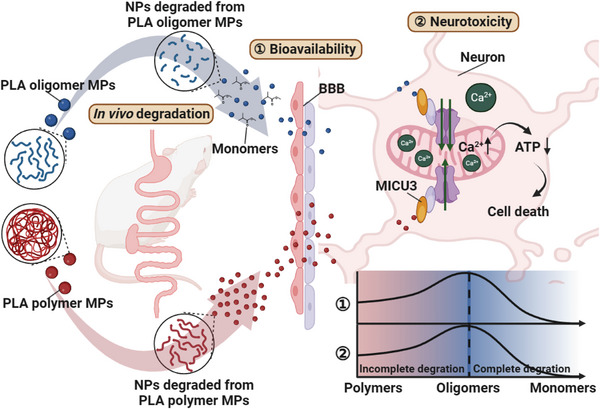
Schematic diagram of the present study. PLA polymer and oligomer MPs undergo incomplete and complete degradation, respectively, in the gastrointestinal tract of mice. The incomplete degradation of PLA polymer MPs heightens their bioavailability by transforming them into oligomer NPs, while the complete degradation of PLA oligomer MPs reduces the bioavailability of PLA particles. Both PLA polymer and oligomer MPs induce PD‐like neurotoxicity in mice by upregulating the expression of *MICU3* in the midbrains, subsequently leading to mitochondrial calcium overload in neurons, with PLA oligomer MPs causing a more significant increase in *MICU* expression. ATP, adenosine triphosphate; BBB, blood–brain barrier; PLA, polylactic acid; *Micu3*, mitochondrial calcium uptake family member 3; MPs, microplastics; NPs, nanoplastics.

Altogether, our study reveals that PLA polymer and oligomer MPs undergo incomplete and complete degradation, respectively, in the gastrointestinal tract of mice. The incomplete degradation of PLA polymer MPs by gastrointestinal digestion heightens their bioavailability and toxicity by transforming into oligomer NPs, exacerbating the overall toxic effects associated with PLA MPs. Conversely, the complete degradation of PLA oligomer MPs by gastrointestinal digestion reduces the bioavailability of PLA particles and weakens their toxicity, ultimately mitigating the overall toxic effects induced by PLA MPs. Additionally, our investigation provides a detailed panorama of the biodistribution of PLA MPs with different molecular weights in mice following in vivo digestion, laying a valuable foundation for subsequent investigations into their toxicity effects and mechanisms. Furthermore, we identify shared targets and toxicity mechanisms in the neurotoxic effects induced by both PLA polymer and PLA oligomer MPs. This reveals that both PLA polymer and oligomer MPs induce PD‐like neurotoxicity in mice by upregulating the expression of MICU3 in the midbrains, subsequently leading to mitochondrial calcium overload in neurons (**Scheme** **1**). Our study advances the understanding of the implications of gastrointestinal degradation on the toxicological impacts of biodegradable MPs on human health, providing critical insights for assessing their risks and benefits compared to their conventional counterparts, and underscoring the urgent and imperative necessity to mitigate human exposure to biodegradable MPs.

## 4 Experimental Section

### Microplastics and Chemicals

The red‐fluorescent polymer and oligomer PLA MPs were obtained from Shanghai Sur‐Release Biotech Inc (Shanghai, China). MCU‐i4 (MCU inhibitor; Glpbio, China) or DBcAMP (NCLX agonist; Glpbio, China) and other chemicals were sourced from commercial suppliers with the highest available purity.

### Particle Characterization

To investigate fluorescence leakage during the digestion process of both types of PLA MPs, simulated gastric and intestinal fluids were prepared using established protocols.^[^ ^27^ ^]^ Fluorescence leakage was assessed by incubating 1 mL suspensions of PLA MPs (0.25 mg mL^−1^) in simulated gastric or intestinal fluids at 37 °C for 1, 2, 3, 4, 5, 6, and 7 days, following previously described methods.^[^ ^41^ ^]^

To investigate the particle characterization of PLA MPs after in vivo digestion, mouse feces were collected on days 1, 14, and 28 of the exposure periods. The feces were weighed, homogenized in double‐distilled water, and filtered through a 70 µm filter, selected for its relatively large pore size to prevent potential aggregation loss of PLA MPs. These feces filtrates were used for the characterization of degraded PLA MPs. Particle surface morphology before and after in vivo degradation was examined using SEM (Zeiss Supra55, Carl Zeiss AG, Germany), following established protocol.^[^ ^42^ ^]^ For the original polymer and oligomer PLA MPs, suspensions were prepared in distilled water at a concentration of 0.25 mg mL^−1^. The size distribution and zeta potential of these suspensions and feces filtrates were determined using DLS with a Zetasizer Nano ZS (Malvern Panalytical GmbH, Kassel, Germany). The infrared spectra of feces filtrates were dried and analyzed using an attenuated total reflection FTIR system (Nicolet 6700 from Thermo Nicolet Corp., Madison, WI) with a scan range of 400 to 4000 cm^−1^. GPC analysis of the PLA MPs was carried out at room temperature using a high‐temperature gel permeation chromatographer (GPC‐220, Agilent, USA). Both pre‐ and post‐digestion PLA MPs were suspended in chloroform and loaded onto the column. Samples were eluted at a flow rate of 1.0 mL min^−1^ with HPLC grade chloroform as the mobile phase, following the established protocol.^[^ ^15^ ^]^

### Ethical Approval

All animal experiments adhered to the National Guidelines for Animal Care and Use of China. The study received approval from the Southern Medical University Scientific Research Committee on Ethics in the Care and Use of Laboratory Animals (Permit No. SMUL202308006).

### Mouse Husbandry

A total of 162 six‐week‐old male C57BL/6 J mice, weighing 20–22 g, were utilized in this study. These mice were procured from the Guangdong Medical Laboratory Animal Center in Guangzhou, China. Upon arrival, a 7‐day acclimatization period was provided to allow the animals to adjust to the new environment. Housing conditions were maintained in a temperature‐controlled room at 23–25 °C with a humidity level of 50–60%. A 12‐h light/dark cycle was enforced (lights on from 8:00 a.m. to 8:00 p.m.). Mice were given access to food and sterilized water ad libitum. Random allocation into specific groups was performed based on their initial body weights at the beginning of the experiment.

### A 28‐Day Repeated Dose Oral Biodistribution and Toxicity Study in Mice

To investigate the biodistribution and neurotoxicity of polymer and oligomer PLA MPs in mice, a 28‐day repeated oral gavage study was conducted involving 114 mice. For the biodistribution study, 24 mice were randomly assigned into three groups: a control group, an oligomer PLA MP group (25 mg kg^−1^), and a polymer PLA MP group (25 mg kg^−1^). For the toxicity study, 90 mice were randomly assigned into five groups: a control group, two oligomer PLA MP groups (2.5 or 25 mg kg^−1^), and two polymer PLA MP groups (2.5 or 25 mg kg^−1^). The mice received daily oral gavage with either double‐distilled water, polymer, or oligomer PLA MPs suspended in double‐distilled water at a dosage of 10 mL kg^−1^ body weight for 28 days. Body weights were assessed every 7 days, and gavage volumes were adjusted weekly based on body weight. All these procedures were conducted between 8:00 a.m. and 9:00 a.m.

At the end of exposure, mice were anesthetized (0.3% pentobarbital sodium, intraperitoneal) and perfused with saline. Tissue samples were then extracted from the euthanized mice on ice. For the determination of polymer and oligomer PLA MP distribution in mice, eight mice (five for tissue homogenate and three for frozen section) in each group were utilized. To measure residual PLA MPs in the intestine, the intestinal contents were not washed. Tissue samples were immediately frozen in liquid nitrogen and stored at −80 °C until use. Five mice in each group were used for qPCR and western blotting detection, and another five mice in each group were used for mitochondrial Ca^2+^ concentration and cellular ATP content detection. The midbrain, hippocampus, striatum, and cortex were collected and separated in half, with tissue samples frozen immediately in liquid nitrogen and stored at −80 °C until use. Three mice in each group were perfused with 4% wt./vol. paraformaldehyde (PFA) after anesthesia, and brains were post‐fixed in 4% PFA at 4 °C overnight for pathological examination.

### Fluorescence Imaging In Vivo

On days 1, 14, and 28 of the gavage periods, three mice in each group were anesthetized with isoflurane prior to gavage on each respective day and underwent in vivo fluorescence imaging using an In Vivo Multispectral FX PRO system (Bruker, Billerica, MA, USA) with excitation at 530 nm and emission at 600 nm.

### MCU‐i4 and DBcAMP Treatment

Forty‐eight mice were randomly divided into three groups (*n* = 16 per group): a control group, an MCU‐i4 treatment group, and a DBcAMP treatment group. In each group, half of the mice received daily oral gavage with PLA polymer MPs suspended in double‐distilled water (25 mg kg^−1^) at a dosage of 10 mL kg^−1^ body weight, while the other half received double‐distilled water for 28 days. For the MCU‐i4 treatment group, mice were treated with 2 mm MCU‐i4 at a dose of 10 mL kg^−1^ body weight every 2 days via intraperitoneal injection. For the DBcAMP treatment group, mice were treated with 2 mM DBcAMP at a dose of 10 mL kg^−1^ body weight every 2 days via intraperitoneal injection. For the control group, mice were treated with the same volume of saline every 2 days via intraperitoneal injection.

### Behavioral Tests

Several behavioral tests were conducted on animals (*n* = 5 per group for each test) at the end of the exposure. 1) Open field test: To assess spontaneous activity, locomotion, and anxiety, each mouse was placed in the center of a square field (60 × 60 × 30 cm, Flyde, China) and allowed to move freely for 5 min. Movement and duration were recorded using a digital CCD camera mounted overhead and analyzed with a maze video tracking system (Chucai Electronic Technology Co., Ltd, China). 2) Elevated plus maze: This maze evaluated anxiety levels. Mice were introduced into the center quadrant of a black Plexiglas cross‐shaped elevated plus maze (arms of 12 cm width × 50 cm length) under dark conditions for 5 min. Locomotion was tracked and recorded using Anymaze software (Stoelting, USA). 3) Rotarod test: Motor and coordination were evaluated by training mice for 5 min at a constant speed of 10 rpm for 3 consecutive days on a rotarod apparatus. During testing, the speed incrementally increased from 4 to 40 rpm over 5 min. The trial ended if the animal fell off the rung or rotated the device 2 consecutive times without attempting to walk on the rung. Motor test data were presented as the mean latency time (3 trials) on the rotarod. 4) Grip strength test: To assess neuromuscular strength, mice were permitted to grip a metal grid with all four limbs. Their tails were gently pulled, and the maximum holding force was recorded with a force transducer while the mice released their grips on the grid. The peak holding strength was digitally recorded and scored as force (N). Each mouse was assessed three times alternately, and the mean strength was recorded. All experiments were conducted between 9:00 and 17:00 in the neurobehavioral laboratory under established optimal conditions.

### Blood‐Brain Barrier (BBB) Permeability Assay

At the end of the 28‐day repeated dose oral toxicity study, five mice from each group were anesthetized with 3% pentobarbital and then administered 2 mL kg^−1^ of 2% w/v Evans blue dye (dissolved in PBS; Sigma‐Aldrich, St. Louis, MO, USA) via retro‐orbital injection. Following a 2‐h circulation of Evans blue, the mice were transcardially perfused with cold PBS until the perfusate from the right atrium became colorless and transparent. Subsequently, the brains were collected and weighed, followed by homogenization in 2 mL of 100% N, N‐dimethylformamide (Sigma‐Aldrich) and an overnight extraction at 37 °C. After centrifugation for 10 min at 2000 rpm min^−1^, the supernatants were collected and measured at 620 nm using a Spark multimode microplate reader (TECAN, Männedorf, Switzerland). Evans blue dye contents were calculated by normalizing the exuded Evans blue dye quantities to the tissue weights.

### Fluorescence Detection in Organ Homogenate

Frozen animal tissues in each group were weighted and homogenized in a digestive solution consisting of 1 g L proteinase K (Python biotech, China), 5 g L^−1^ SDS, 23 g L^−1^ Na_2_HPO_4_, and 4.6 g L^−1^ NaH_2_PO_4_ using a high‐speed low‐temperature tissue homogenizer (Servicebio, China) at 60 Hz for 3 min at 4 °C. A volume of 2.5 mL of digestive solution per gram of tissue was used for tissue digestion. The homogenate was digested in a water bath at 37 °C for 12 h, resulting in a clear‐digested tissue solution.

The digested solution of each tissue of mice in the control group was mixed and diluted with double‐distilled water as needed. For intestines, the digested solution was diluted 20 times, and for other organs and blood, the digested solution was diluted 10 times. Serial dilutions of polymer and oligomer PLA MPs in each tissue solution and in the blood were prepared and measured by a fluorescence spectrophotometer (Tecan Spark, Austria) with an excitation wavelength of 538 nm and an emission wavelength of 584 nm. A standard curve for polymer or oligomer PLA MPs in each tissue and the blood was calculated in the corresponding organ homogenate of the control group animals. The digested solution of each tissue and the blood in the exposure group were diluted similarly to the control group, with double‐distilled water, and measured under the same conditions.

After 28 days of exposure, the polymer and oligomer PLA MPs biodistribution were assessed by the concentrations of the polymer or oligomer PLA MPs in each organ or blood, calculated as (particle weight) / (organ weight). The bioavailability of polymer or oligomer PLA MPs was evaluated by the fraction (%) of administered particles that reached the major organs, including the brain, heart, lungs, liver, spleen, kidneys, reproductive organs, and blood, excluding the gastrointestinal tract. It was calculated as (total amount in the major organs and blood) / (total amount of gavage). The accumulation of polymer or oligomer PLA MPs in the intestines after 24 h of exposure was assessed by the fraction (%) of residue particles remaining in the gastrointestinal tract, calculated as (total amount in the gastrointestinal tract) / (total amount of gavage).

### Fluorescent Particle Detection on Tissue Slides

Frozen brains from each group underwent coronal sections for fluorescence detection following the previously protocols.^[^ ^27^ ^]^ The slides were scanned using Pannoramic MIDI (3D HISTECH, Budapest, Hungary), and the images were reviewed with Pannoramic Viewer (3D HISTECH).

### Immunofluorescence Analysis

After deparaffinization and rehydration, slides were blocked with 3% BSA (Servicebio) for 30 min at room temperature. Subsequently, slides were incubated overnight with anti‐tyrosine hydroxylase (TH) mouse monoclonal antibody (GB12181, Servicebio). Following three washes with PBS (pH 7.4), slides were incubated with a secondary antibody against mouse (conjugated with cyanine3) IgG. The detailed information of the antibodies is provided in Table S2 (Supporting Information). The slides were scanned using Pannoramic MIDI after mounting coverslips with 4′,6‐diamidino‐2‐phenylindole (DAPI) solution (Servicebio), and the images were reviewed with Pannoramic Viewer. Fluorescent intensity in each image was measured using ImageJ 1.52 v (National Institute of Health, Bethesda, MD, USA). To quantify the percentage of TH‐positive cell count, TH‐positive cells were counted in each view and normalized to a DAPI count. For each brain section, images (*n* = 3 per target brain region) were acquired for quantification.

### Nissl Staining

After deparaffinization and rehydration, the slides were stained with toluidine blue. Subsequently, images were acquired using an ortho‐fluorescent microscope (Nikon Eclipse C1, Tokyo, Japan). The counting of Nissl‐positive neurons in the cortex, hippocampus, SNc, and striatum regions was performed. To quantify the percentage of Nissl‐positive cell count, Nissl‐positive cells were counted in each view and normalized to a DAPI count. For each brain section, images (*n* = 3 per target brain region) were acquired for quantification.

### TUNEL Fluorescence Assay on Tissue Slides

The in situ TUNEL fluorescence assay was conducted using a Click‐iT TUNEL Alexa Fluor 488 Imaging Assay (Invitrogen) following the manufacturer's instructions. Subsequently, the slides were promptly scanned using Pannoramic MIDI, and the images were examined using Pannoramic Viewer. To quantify the percentage of TUNEL‐positive cell count, TUNEL‐positive cells were counted in each view and normalized to a DAPI count. For each brain section, images (*n* = 3 per target brain region) were acquired for quantification.

### RNA Isolation and qPCR Analysis

Total RNA was isolated from frozen animal tissues using an RNA extraction kit (Agbio, China). For qPCR analysis, mRNA cDNA was synthesized with the HiScript II First Strand cDNA Synthesis Kit (Vazyme, Nanjing, China) following the manufacturer's recommended protocol. mRNA primers (Table S3, Supporting Information) were synthesized by Tsingke Biotechnology (Beijing, China). qPCR was conducted using the SYBR Green Premix Pro Taq HS qPCR kit (Accurate Biotechnology) on the ABI QuantStudio 6 flex. The relative expression of mRNA was normalized to *β‐actin*. Relative gene expression was determined using the 2^−ΔΔCT^ method compared to the control group.

### Bulk RNA‐Seq

Strand‐specific libraries were prepared using the TruSeq Stranded Total RNA Sample Preparation Kit (Illumina, San Diego, CA, USA) following the manufacturer's instructions. Sequencing read counts were calculated with Stringtie (v. 1.3.0). The expression levels from different samples were then normalized using the trimmed mean of M values method.^[^ ^43^ ^]^ Normalized expression levels were then converted into fragments per kilobase of transcript per million mapped fragments (FPKM). The difference in gene expression among groups was analyzed, and *P*‐values were calculated, along with multiple hypothesis tests using the edgeR (v. 3.32.1) package^[^ ^44^ ^]^ in R. GO and GSEA were performed using the clusterProfiler (v. 4.0) package^[^ ^45^ ^]^ in R with enrichment criteria of false discovery rate (FDR) ≤ 0.1 and *p*‐value ≤ 0.05.

### Protein Extraction and Western Blotting

Mouse brain tissue was lysed in RIPA buffer (Beyotime, Shanghai, China), supplemented with a 1:100 dilution of protease and phosphatase inhibitor cocktail (Keygen Nanjing, China), and homogenized for 3 min at 60 Hz using a high‐speed low‐temperature tissue homogenizer (Servicebio). This ensured complete disruption of tissue structure and efficient protein extraction. Protein samples were fractionated by sodium lauryl sulfate‐polyacrylamide gel electrophoresis and transferred to a polyvinylidene fluoride membrane (Bio‐Rad Laboratory, USA). The membranes were blocked with 5% skim milk and probed with primary antibodies overnight at 4 °C (see Table S2, Supporting Information). After washing the membrane three times, it was incubated with HRP‐labeled secondary antibodies for 1 hour at room temperature. Protein bands were revealed by Western Lightning plus‐ECL (PerkinElmer, Waltham, MA, USA), and the grayscale of the bands was quantified using ImageJ software.

### Determining Cellular ATP Content

Fresh brain cellular ATP contents were determined using Enhanced ATP Assay Kits (Beyotime) following the manufacturer's protocol. Briefly, brain tissues were lysed with ATP lysis buffer (100 µL of lysate per 20 mg) and homogenized using a high‐speed low‐temperature tissue homogenizer for 3 min at 60 Hz, followed by centrifugation at 15 000 g for 10 min at 4 °C. The supernatants were collected, and 20 µL of each supernatant and ATP standards were added to wells containing 100 µL ATP detection working dilution in 96‐well plates. Luminescence was detected with a Spark multimode microplate reader (TECAN). ATP content was calculated using an ATP standard curve and normalized by protein content.

### Mitochondrial Calcium Determination

Fresh brain tissues were homogenized in mitochondrial isolate lysis (Beyotime) using a high‐speed low‐temperature tissue homogenizer for 15 s at 60 Hz. After centrifugation at 4 °C, 2,000 g for 3 min, the pellet was resuspended in 1 mL of mitochondrial isolate and centrifuged again at 4 °C, 2,000 g for 3 min. The supernatant obtained from the two centrifugations was mixed and centrifuged at 14 000 g for 8 min, and the supernatant was discarded, leaving the precipitate as the isolated mitochondria. The isolated mitochondria underwent spectrophotometric determination of Ca^2+^ content using the Calcium Colorimetric Assay Kit (Beyotime).

### Cell Culture and siRNA‐Mediated Interference

SH‐SY5Y cells were maintained at 37 °C in a 5% CO_2_ atmosphere in Dulbecco's Minimum Essential Medium (DMEM) supplemented with 10% fetal bovine serum (FBS) and 1% antibiotics. The cells were cultured in Petri dishes and passaged when they reached 75–85% confluency, using 0.25% Trypsin‐EDTA for cell detachment. SH‐SY5Y cells were differentiated with 10 µm All trans‐retinoic acid for 3 days followed by 80 nm 12‐o‐tetradecanoylphorbol‐13‐acetate for another 3 days. The differentiated SH‐SY5Y cells were seeded into 6‐well plates at a density of 3 × 10^5^ cells per well and incubated overnight. Subsequently, these cells were treated with PLA MPs for 24 h. Following incubation, the cells were washed three times with PBS to eliminate any adherent PLA MPs prior to further analysis.

To silence gene expression, 100 nm siRNA was transfected into the SH‐SY5Y cells using standard procedures with Lipofectamine RNAiMAX Transfection Reagent (Tsingke) according to the manufacturer's instructions. Cells were stimulated and harvested for further analysis 48 h after transfection. The following siRNA sequences were used: human si‐*Micu3*‐1 sense, 5′‐GCGUUACAGUACCUGAA‐3′, human si‐*Micu3*‐1 antisense, 5′‐UUCAGGUAUACUGUAACGC‐3′; human si‐*Micu3*‐2 sense, 5′‐GGGAUACACUGAGACGUAA −3′; human si‐*Micu3*‐2 antisense, 5′‐UUACGUCUCAGUGUAUCCC‐3′.

### Statistical Analysis

Statistical analyses were conducted using R^[^ ^46^ ^]^ (v. 4.0.2) and SPSS 22.0 (IBM, Armonk, NY, USA). The data are presented as mean ± standard deviation (SD) unless otherwise specified. To detect statistical significance in differences between the two groups, a Student's *t*‐test was applied. For comparisons involving multiple groups, one‐way analysis of variance (ANOVA) was performed, followed by a Tukey's multiple comparisons test, unless otherwise specified. Statistical significance was determined by a two‐sided probability (*p*) < 0.05.

## Conflict of Interest

The authors declare no conflict of interest.

## Author Contributions

B.L., Y.D., and Y.Z. contributed equally to this work as co‐first authors. B.L., Y.D., and Y.Z. performed conceptualization, methodology, visualization, data curation, formal analysis, and writing – original draft. X. C., X.H., and X.Y. performed in vitro experiments and data analysis. Y.H., Z.L., J.D., R.Y., and H.X. performed in vivo experiments and data analysis. Y.F., R.B., and B.F. performed characterization experiments and data analysis. X.Y. performed project administration. Z.H. performed conceptualization, resources, funding acquisition, project administration, and writing – review and editing.

## Supporting information

Supporting Information

## Data Availability

The data that support the findings of this study are available from the corresponding author upon reasonable request.
