# Supplementary material for: Gastrointestinal Incomplete Degradation Exacerbates Neurotoxic Effects of PLA Microplastics via Oligomer Nanoplastics Formation
Source: Adv Sci (Weinh). 2024 May 15;11(28):2401009. doi: 10.1002/advs.202401009 (PMC11267364; doi:10.1002/advs.202401009)
Supplement: Supplementary file 1 — Supporting Information [file ADVS-11-2401009-s001.pdf]

ADVANCED SCIENCE
Open Access

Supporting Information

for *Adv. Sci.*, DOI 10.1002/advs.202401009

Gastrointestinal Incomplete Degradation Exacerbates Neurotoxic Effects of PLA Microplastics via Oligomer Nanoplastics Formation

*Boxuan Liang, Yanhong Deng, Yizhou Zhong, Xiaoqing Chen, Yuji Huang, Zhiming Li, Xiyun Huang, Xiaohong Yang, Jiaxin Du, Rongyi Ye, Hongyi Xian, Yu Feng, Ruobing Bai, Bingchi Fan, Xingfen Yang and Zhenlie Huang**

Supporting Information

Gastrointestinal Incomplete Degradation Exacerbates Neurotoxic Effects of PLA Microplastics via Oligomer Nanoplastics Formation

Boxuan Liang [1,2,*], Yanhong Deng [2,*], Yizhou Zhong [2,*], Xiaoqing Chen [2], Yuji Huang [2], Zhiming Li [2], Xiyun Huang [2], Xiaohong Yang [2], Jiaxin Du [2], Rongyi Ye [2], Hongyi Xian [2], Yu Feng [2], Ruobing Bai [2], Bingchi Fan [2], Xingfen Yang [3], and Zhenlie Huang [2,4**]

[1] The Tenth Affiliated Hospital, Southern Medical University (Dongguan People's Hospital), Dongguan 523059, China;

[2] NMPA Key Laboratory for Safety Evaluation of Cosmetics, Guangdong Provincial Key Laboratory of Tropical Disease Research, School of Public Health, Southern Medical University, Guangzhou 510515, China;

[3] NMPA Key Laboratory for Safety Evaluation of Cosmetics, Guangdong Provincial Key Laboratory of Tropical Disease Research, Research Center for Food safety and Health, School of Public Health, Southern Medical University, Guangzhou 510515, China;

[4] Department of Cardiovascular Surgery, Zhujiang Hospital, Southern Medical University, Guangzhou 510280, China.

*These authors contributed equally to this work as co-first authors.

**Corresponding author (*Zhenlie Huang*) E-mail: huangzhenlie858252@smu.edu.cn

ORCID: 0000-0001-9818-8192

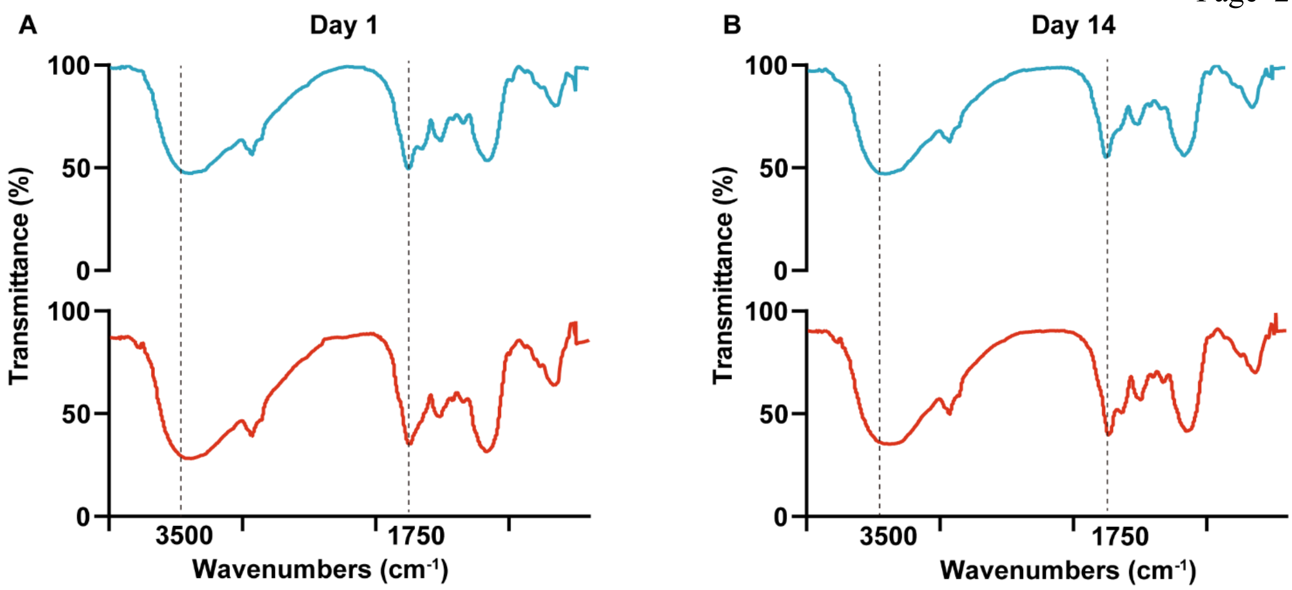

Figure S1. Fourier-transform infrared spectroscopy spectra of polylactic acid oligomer and polymer microplastics after *in vivo* digestion. **A**) Spectra after 1 day of digestion. **B**) Spectra after 14 days of digestion.

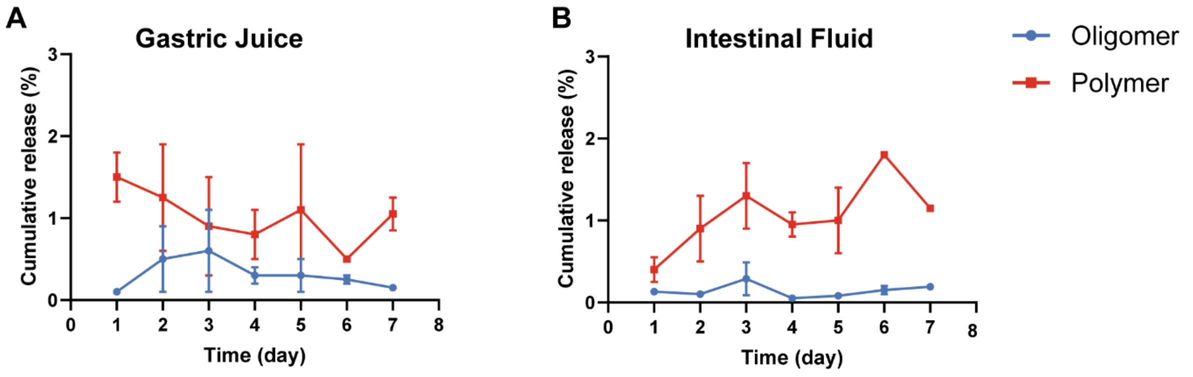

Figure S2. Fluorescence leakage from polylactic acid oligomer and polymer microplastics incubated in **A**) gastric juice and **B**) intestine juice. Fluorescence leakage values are presented as means ± SD (*n* = 3 per group).

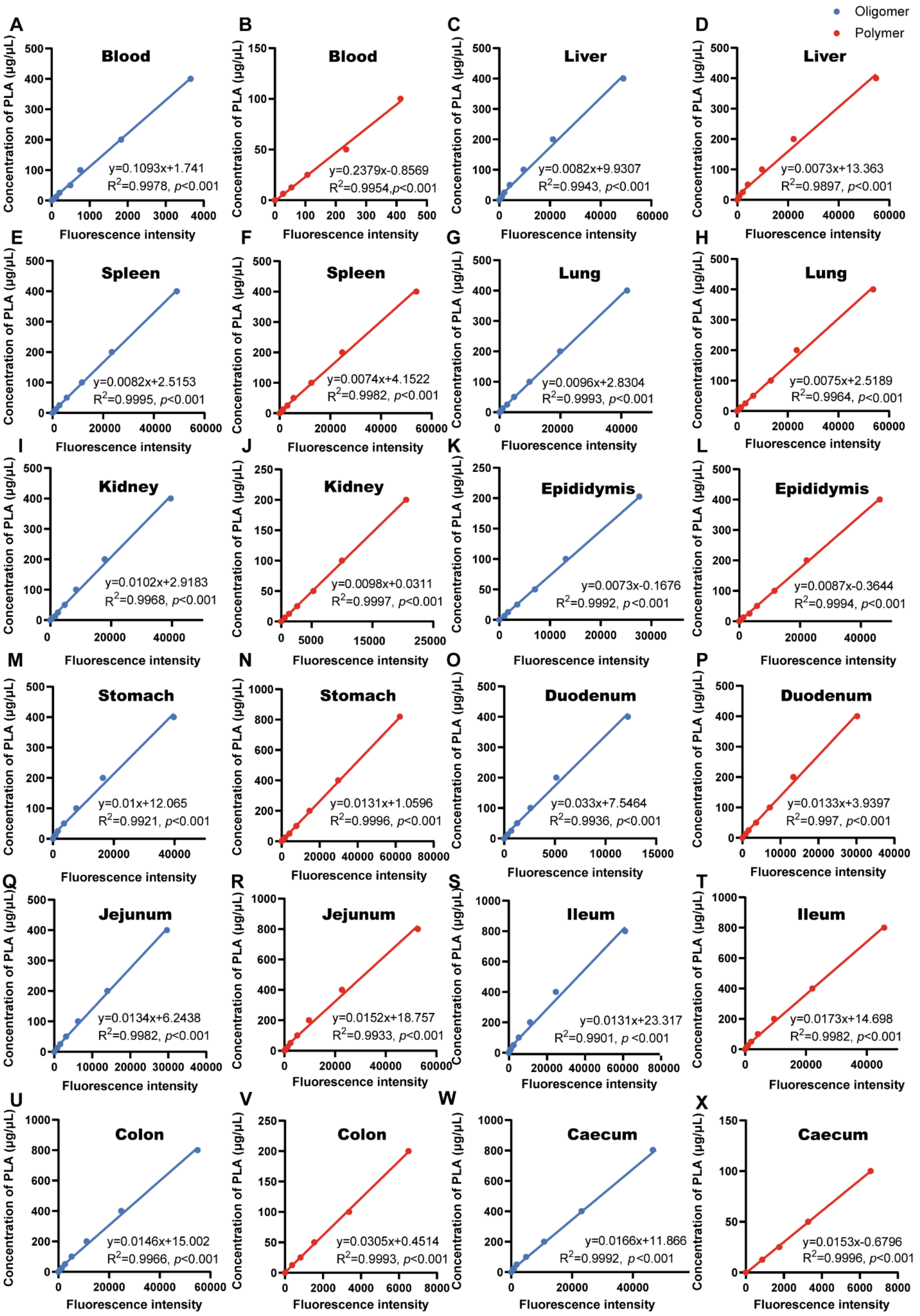

Figure S3. Standard curves of fluorescence intensity of polylactic acid oligomer and polymer microplastics in A and B) blood, C and D) liver, E and F) spleen, G and H) lung, I and J) kidney, K

and L) epididymis, M and N) stomach, O and P) duodenum, Q and R) jejunum, S and T) ileum, U and V) colon, W and X) caecum.

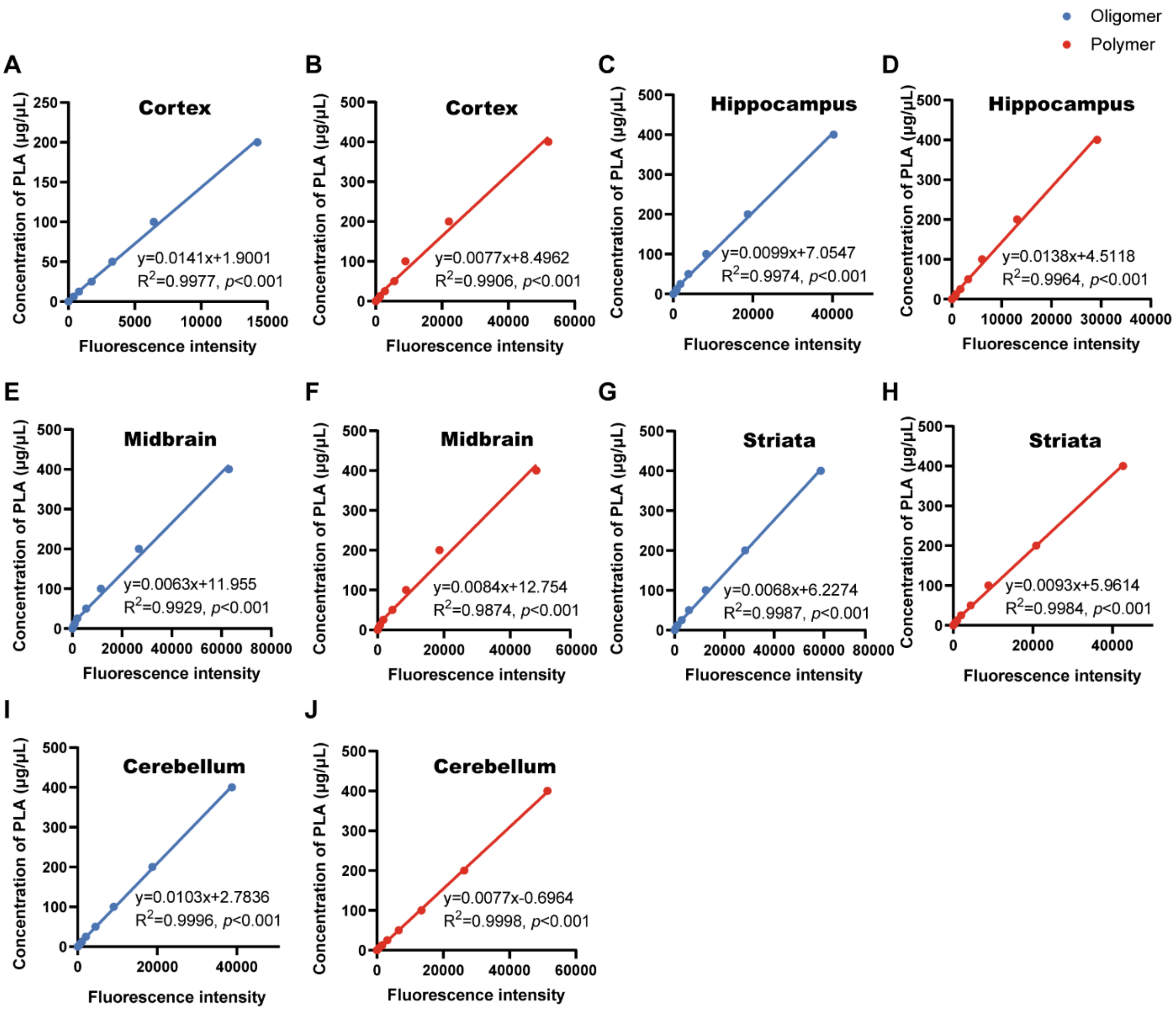

Figure S4. Standard curves of fluorescence intensity of polylactic acid oligomer and polymer microplastics in A and B) cortex, C and D) hippocampus, E and F) midbrain, G and H) striata, I and J) cerebellum.

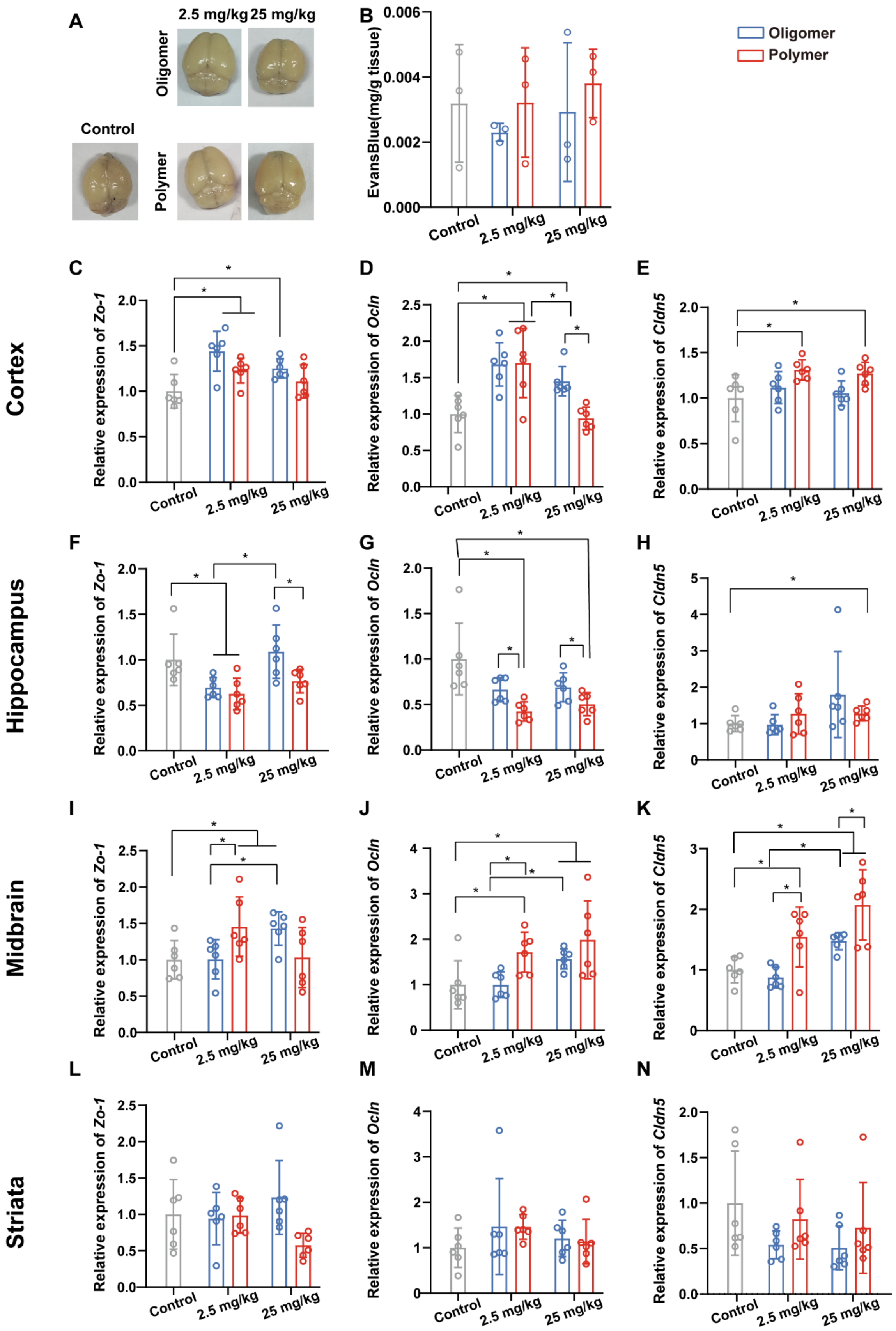

Figure S5. Effect of exposure to polylactic acid oligomer and polymer microplastics on the BBB. **A**) Representative images and **B**) quantification of brains after Evans blue staining. **C-E**) Expression of

BBB-related genes in the cortex. **F-H**) Expression of BBB-related genes in the hippocampus. **I-K**) Expression of BBB-related genes in the midbrain. **L-N**) Expression of BBB-related genes in the striatum. BBB, blood-brain barrier.

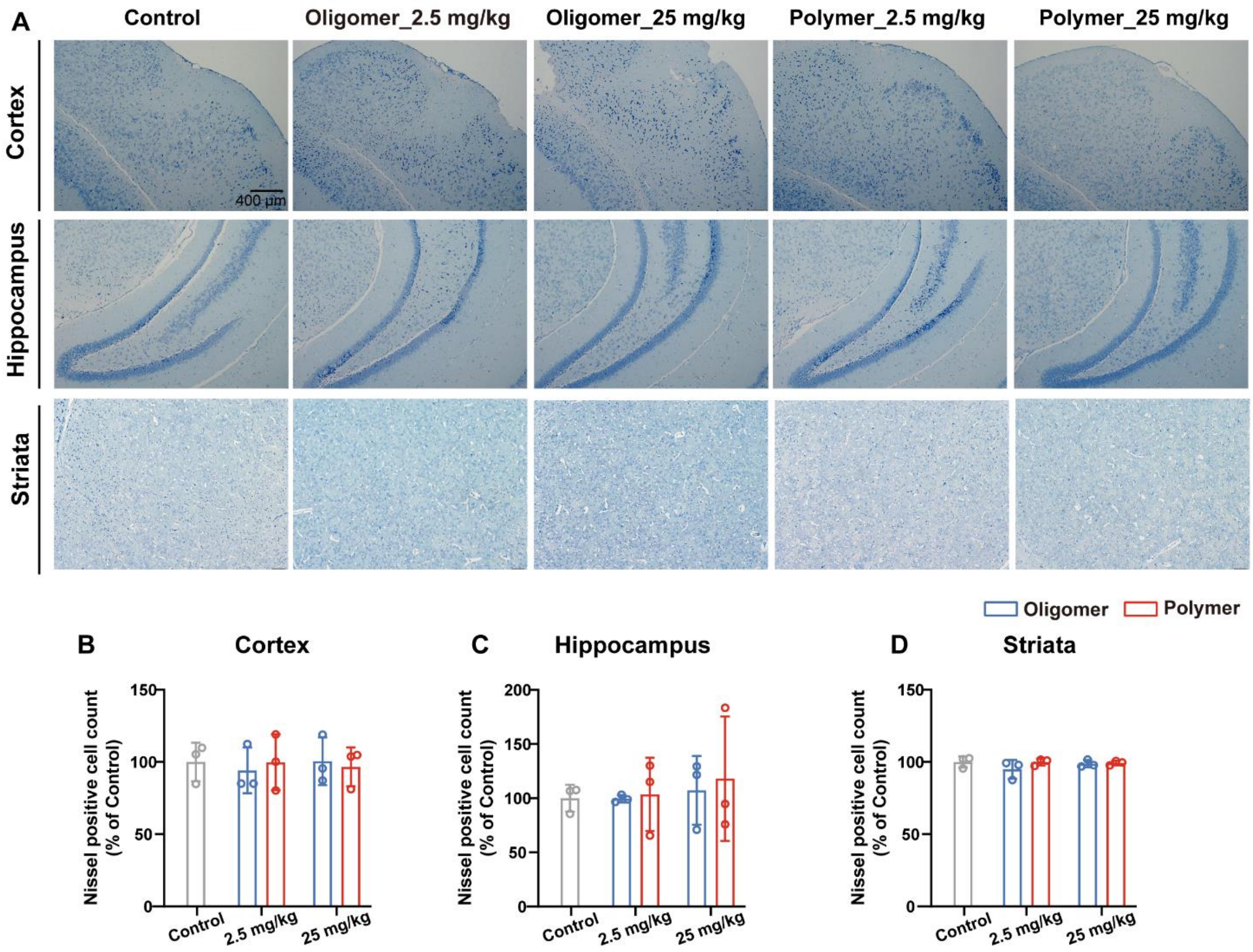

Figure S6. Nissl staining of cortex, hippocampus and striata regions after exposure to polylactic acid oligomers and polymeric microplastics. **A**) Representative Nissl staining images from mouse cortex, hippocampus and striata. Relative percentages of positive Nissl staining cells in **B**) cortex, **C**) hippocampus and **D**) striata. Statistical analyses are determined by ANOVA, followed by Tukey's multiple comparison tests. *$P < 0.05$. ANOVA, analysis of variance.

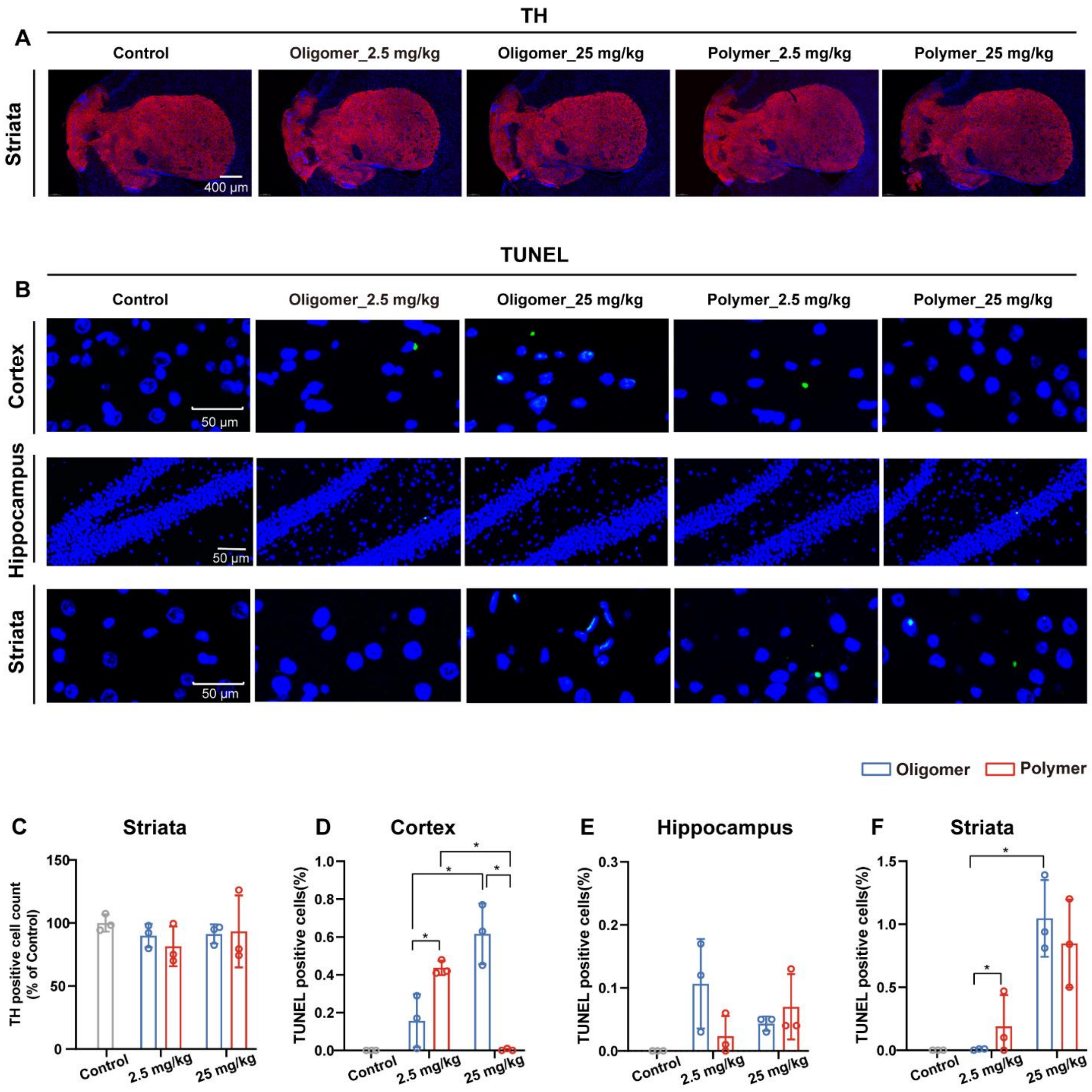

Figure S7. TH and TUNEL staining after exposure to polylactic acid oligomers and polymeric microplastics. **A**) Representative TH staining images from mouse striata. **B**) Representative TUNEL staining images from mouse cortex, hippocampus and striata. **C**) Relative percentages of positive TH staining cells in striata. Relative percentages of positive TUNEL staining cells in **D**) cortex, **E**) hippocampus and **F**) striata. Statistical analyses are determined by ANOVA, followed by Tukey's multiple comparison tests. *$P < 0.05$. ANOVA, analysis of variance; TH, tyrosine hydroxylase; TUNEL, TdT-mediated dUTP nick-end labeling.

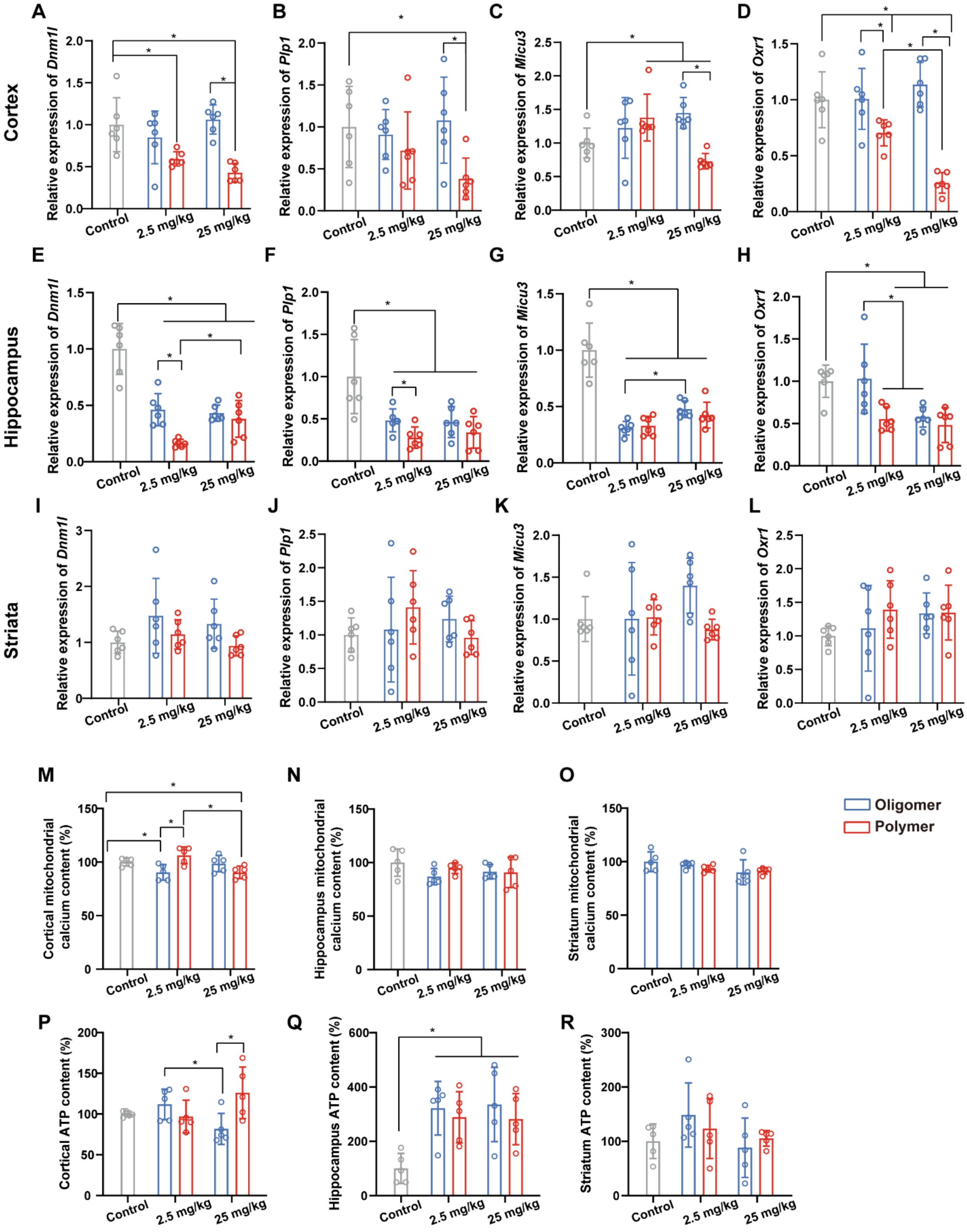

Figure S8. Expression of calcium ion transport related genes in the **A-D**) cortexes, **E-H**) hippocampi, and **I-L**) striata. Calcium concentration in the **M**) cortexes, **N**) hippocampi, and **O**) striata. ATP content in the **P**) cortexes, **Q**) hippocampi, and **R**) striata. Statistical analyses are

determined by ANOVA, followed by Tukey's multiple comparison tests. *$P < 0.05$. ANOVA, analysis of variance.

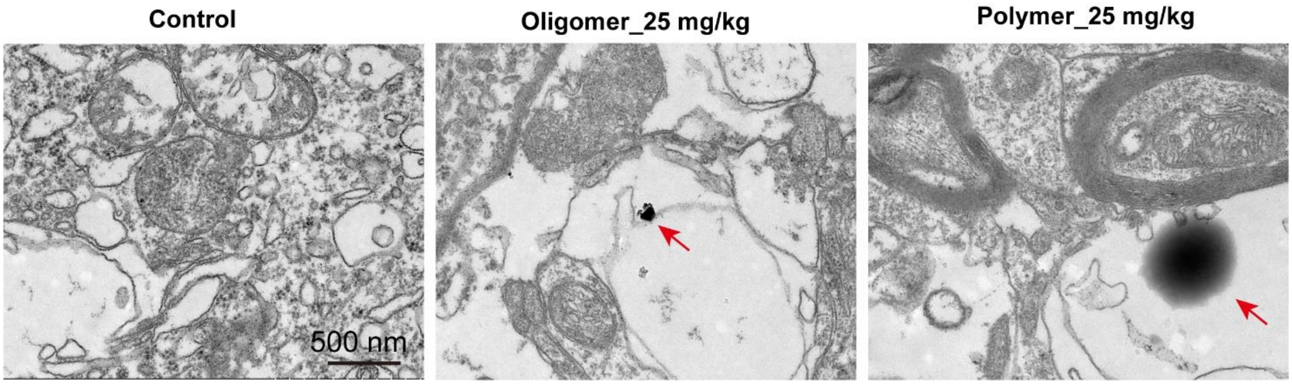

Figure S9. Representative images of transmission electron microscope in mouse midbrain substantia nigra, the red arrows indicate mitochondria.

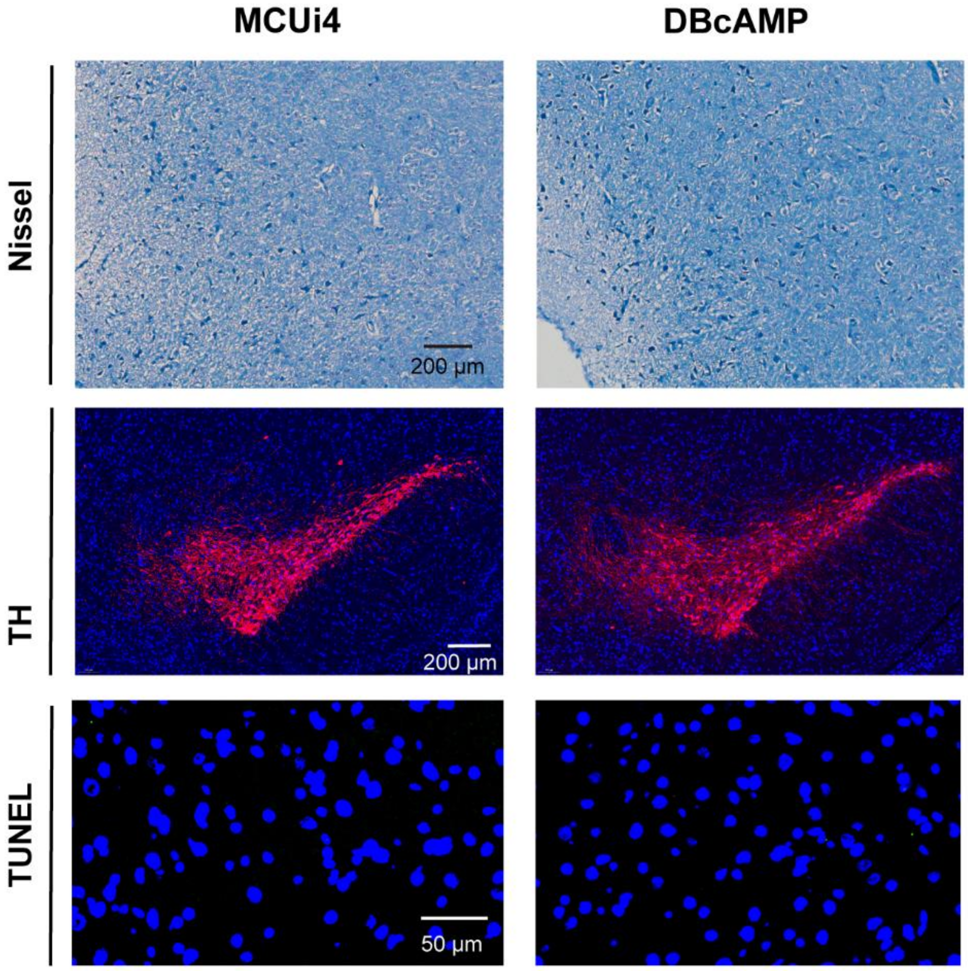

Figure S10. Representative images of Nissl staining, tyrosine hydroxylase staining and TdT-mediated dUTP nick-end labeling staining in mouse midbrains after MCU-i4 or DBcAMP treatment.

Table S1. The body weight and coefficients of major organs in each experimental group.

Group	Control	Oligomer_2.5 mg/kg	Oligomer_25 mg/kg	Polymer_2.5 mg/kg	Polymer_25 mg/kg
Body weight (g)	23.5 ± 2	24.8 ± 1.1	22.7 ± 1.3	24.9 ± 1.1	24.7 ± 1.5
Brain Coefficient (%)	1.68 ± 0.13	1.58 ± 0.04	1.76 ± 0.11	1.61 ± 0.06	1.68 ± 0.10
Heart Coefficient (%)	0.47 ± 0.04	0.5 ± 0.08	0.46 ± 0.03	0.47 ± 0.04	0.48 ± 0.04
Liver Coefficient (%)	5.34 ± 0.46	5.49 ± 0.19	5.13 ± 0.30	5.08 ± 0.38	5.41 ± 1.32
Spleen Coefficient (%)	0.30 ± 0.05	0.3 ± 0.04	0.30 ± 0.09	0.25 ± 0.03	0.28 ± 0.07
Lung Coefficient (%)	0.71 ± 0.25	0.56 ± 0.04	0.70 ± 0.30	0.54 ± 0.08	0.91 ± 0.31
Kidney Coefficient (%)	1.18 ± 0.17	1.26 ± 0.09	1.19 ± 0.15	1.26 ± 0.10	1.21 ± 0.14
Epididymis Coefficient (%)	0.17 ± 0.06	0.17 ± 0.11	0.21 ± 0.08	0.17 ± 0.09	0.20 ± 0.05
Testis Coefficient (%)	0.24 ± 0.06	0.32 ± 0.03	0.24 ± 0.09	0.30 ± 0.04	0.25 ± 0.05

n = 10 in each group.

Table S2. List of antibodies for immunofluorescence and western blotting.

Primary antibody	Manufacturer	Catalogue	Dilution	Second antibody	Manufacturer	Catalogue	Dilution
TH	Servicebio	GB12181	1:500	Cyanine3 conjugated anti-rabbit	Servicebio	GB22401	1:200
MICU3	Abcepta	AI13855	1:1000	HRP-conjugated Affinipure Goat Anti-Rabbit IgG	Proteintech	SA00001	1:5000
GAPDH	Proteintech	60004-1-Ig	1:1000	HRP-conjugated Affinipure Goat Anti-Mouse IgG	Proteintech	PR30012	1:5000

Table S3. Primer sequences for qPCR.

Gene	GenBank accession	Forward Primer (5'-3')	Reverse Primer (5'-3')
Micu3	NM_030110	CCCAAATTTGCTAAAACGTGGA	AAACCTGCATGGGGCTTTGT
Oxr1	NM_001130163	GCAAACCCTGGAAAACTCACTCT	CATCGTAGCACTTGCTTGCG
Plp1	NM_001290561	CTGGCTGAGGGCTTCTACAC	GTGATGCCCACAAACGTTGC
Dnm1l	NM_001025947	GTAGTGGGAACGCAGAGCAG	ACCCCATTCTTCTGCTTCAAC
Ocln	NM_001360536	TGAAAGTCCACCTCCTTACAGA	CCGGATAAAAAGAGTACGCTGG
Cldn5	NM_013805	GCAAGGTGTATGAATCTGTGCT	GTCAAGGTAACAAAGAGTGCCA
Zo-1	NM_001163574	GCCGCTAAGAGCACAGCAA	GCCCTCCTTTTAACACATCAGA
β-actin	NM_007393	GGCTGTATTCCCCTCCATCG	CCAGTTGGTAACAATGCCATGT
MICU3	NM_001349810	AGATGAGCCCAAAGTTGCCA	GAACCCTGCATGTGGCTTTG
β-ACTIN	NM_001101	CATGTACGTTGCTATCCAGGC	CTCCTTAATGTCACGCACGAT

qPCR, quantitative polymerase chain reaction.
